# Hyper-Responsive Chemiluminescent Probe Reveals Distinct PYRase Activity in *Pseudomonas aeruginosa*

**DOI:** 10.1021/acs.bioconjchem.4c00015

**Published:** 2024-03-22

**Authors:** Rozan Tannous, Omri Shelef, Tal Kopp, Micha Fridman, Doron Shabat

**Affiliations:** † School of Chemistry, Raymond and Beverly Sackler Faculty of Exact Sciences, Tel-Aviv University, Tel Aviv 69978, Israel

## Abstract

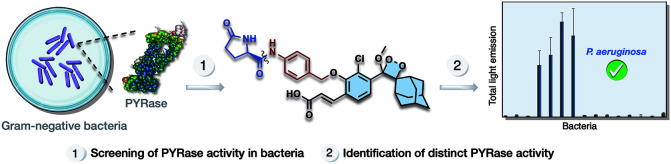

Pyrrolidone carboxyl peptidase, commonly known as PYRase, is an exopeptidase that catalytically cleaves an *N*-terminal pyroglutamic acid from peptides or proteins. The diverse functions of PYRases in bacterial enzymology have prompted the development of various bacterial diagnostic techniques. However, the specific physiological role and activity of this enzyme across the bacterial kingdom remain unclear. Here, we present a functional phenoxy-1,2-dioxetane chemiluminescent probe (PyrCL) that can selectively detect PYRase activity in both Gram-positive and Gram-negative bacteria. The probe activation mechanism is based on the cleavage of a pyroglutamyl substrate, followed by a release of the phenoxy-dioxetane luminophore, which then undergoes efficient chemiexcitation to emit a green photon. Probe PyrCL exhibits an effective turn-on response with superior detection capability in terms of response time and sensitivity compared to existing fluorescence probes. The superior detection sensitivity of the chemiluminescent probe enables us to reveal previously undetected PYRase activity in *Streptococcus mutans*. Furthermore, it enables the discrimination of *Pseudomonas aeruginosa* from other Gram-negative bacteria in the tested panel, based on their distinct PYRase activity. We expect that probe PyrCL will have great value for PYRase-based bacteria diagnosis with use in basic research and clinical applications.

Pyrrolidone carboxyl peptidase, also known as pyroglutamyl peptidase, is an exopeptidase commonly referred to as PYRase.^1^ This enzyme plays a pivotal role in the hydrolytic removal of the *N*-terminal pyroglutamic acid (Pyr) from Pyr-peptides or Pyr-proteins. Since its discovery over four decades ago, PYRase activity has been observed in various organisms, including bacteria, plants, animals, and human tissues.^2^ Interestingly, bacterial PYRases display many of the biochemical characteristics common to mammalian PYRases, such as a broad Pyr substrate specificity and a strict requirement for a highly reduced environment.^3^ The diverse functions of PYRases in bacterial enzymology have prompted the development of various bacterial diagnostic techniques, relying on specific chromogenic and fluorogenic substrates.^4,5^ In 1969, Mulczyk and Szewczuk reported the first probe system assay for the detection of PYRase activity.^6^ The assay was subsequently commercialized and since then has been the most widely used PYRase probe.^7−9^ In this two-step colorimetric assay, Pyr-β-naphthylamide is initially incubated for 3 h with the sampled bacteria. In the first step, enzymatic removal of the Pyr group by PYRase results in the release of free β-naphthylamine. In the second step, the β-naphthylamine is subjected to a coupling reaction with tetra-azotized *o*-dianisidine (Fast Blue B) in acetic acid, for 1 h, to generate an observable red dye. This assay has a laborious protocol that requires up to 4 h to produce a positive indication. To circumvent this laborious procedure, a fluorescent-based assay was later developed.^10^ The assay relies on the release of the 7-amino-4-methyl-coumarin (AMC) dye through bacterial PYRase activity, utilizing the probe Pyr-AMC. Unlike the Pyr-β-naphthylamide test, the assay involving the probe Pyr-AMC occurs through a single step, requires no additional reagents, and can rapidly provide a positive indication. Despite this advancement in the detection of PYRase activity, the specific physiological role and activity of this enzyme across the bacterial kingdom remain unclear.^1^

Chemiluminescent probes have emerged as versatile and powerful molecular tools for diagnostic and imaging applications.^11^ Unlike fluorescence, chemiluminescence involves the emission of light resulting from a chemical reaction without the requirement of an external light source. This inherent advantage eliminates background interference since autofluorescence and light scattering do not occur.^12^ As such chemiluminescent probes are among the most sensitive tools currently employed to detect enzymatic activity.^13^ In recent years, the development of chemiluminescent probe technologies was significantly advanced by the discovery of *ortho*-substituted phenoxy-dioxetanes.^14^ The incorporation of an acrylate substituent at the *ortho* position of a phenoxy-adamantyl-1,2-dioxetane, prevents water-mediated quenching of the excited intermediate, enabling up to 3000-fold enhancement of light-emission intensity. These probes found widespread applications in bioimaging, immunoassays, and real-time monitoring of cellular events both *in vitro* and *in vivo*.^15−28^ Here, we report a highly sensitive chemiluminescent probe for the detection of PYRase and its use for the rapid screening of its activity across numerous Gram-positive and Gram-negative bacterial strains.

The molecular structure of probe PyrCL and its chemiluminescent activation pathway are illustrated in Figure 1A. The design of probe PyrCL comprises three key components: a pyroglutamyl substrate, a *para*-amino benzyl alcohol self-immolative spacer, and a phenoxy 1,2-adamantylidene-dioxetane luminophore bearing an acrylic acid substituent at the *ortho*-position. Enzymatic cleavage of the pyroglutamyl substrate by PYRase, followed by 1,6-elimination of azaquinone methide, leads to the release of phenoxy-1,2-dioxetane. The latter undergoes chemiexcitation, which results in the emission of a green photon.

**Figure 1:**
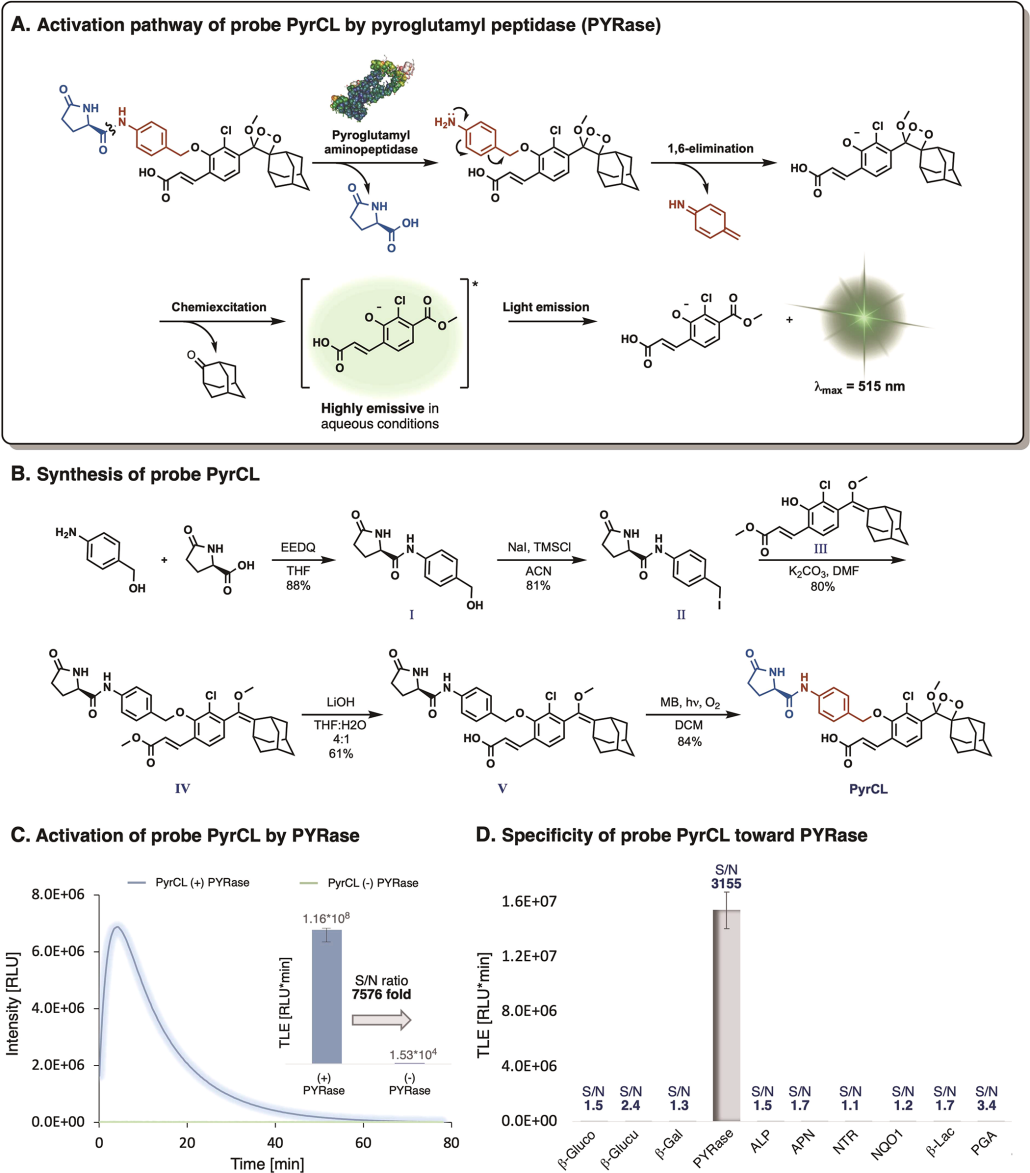
Probe PyrCL (A) Chemiluminescent activation pathway, (B) synthesis, (C) activation by the commercial enzyme PYRase, and (D) specificity in the presence of common bacterial enzymes. Detailed procedures are described in the Supporting Information. Error bars represent the ±SD of three independent measurements.

The synthesis of the PyrCL probe was achieved as described in Figure 1B. *para*-Aminobenzyl-alcohol was coupled with pyroglutamic acid to form amide **I**. The latter was then treated with sodium iodide and trimethylsilyl chloride to produce benzyl-iodide **II**. Nucleophilic substitution of benzyl-iodide **II** by the previously synthesized phenol enolether **III** afforded enolether **IV**. The methyl ester group of enolether **IV** was then hydrolyzed using lithium hydroxide and water to give carboxylic acid-enolether **V**, which was subsequently oxidized by singlet oxygen to yield probe PyrCL.

Initially, we sought to evaluate the light-emission turn-on response of probe PyrCL upon reaction with PYRase. The chemiluminescent kinetic profile of probe PyrCL incubated in the presence and absence of PYRase under physiological conditions (PBS, pH 7.4) and the total light emission values are presented in Figure 1C. Probe PyrCL exhibits a typical chemiluminescence kinetic profile upon incubation with PYRase, with an initial light-emission increase to a maximum, followed by decay of the signal over 60 min. The total light emission measured by probe PyrCL in the presence of PYRase was 7576-fold greater than that observed in the absence of the enzyme. We then investigated the specificity of probe PyrCL for its target enzyme PYRase, assessing it across 10 commercially available common bacterial enzymes (Figure 1D). Probe PyrCL exhibited superior specificity as a substrate for detecting PYRase activity, resulting in an intense light emission signal with an S/N value of 3155-fold. This S/N value was about 3 orders of magnitude higher than S/N values obtained in the presence of all other enzymes (S/N values of 1.1–3.4).

We then aimed to compare the detection sensitivity of probe PyrCL toward PYRase activity with that of a commercially available fluorescent probe. Probe Pyr-AMC, the most commonly used fluorescent pyroglutamyl substrate, is composed of aminomethylcoumarin (AMC) masked with pyroglutamic amino acid. The structure of the chemiluminescent probe, PyrCL, features an additional self-immolative spacer conjugated to the pyroglutamyl substrate in comparison to the Pyr-AMC probe. Since previous studies have shown that addition of the self-immolative spacer, between the substrate and the fluorescence dye, improves the probe’s enzymatic substrate recognition, we also used probe Pyr-HC for this comparison evaluation. Probe Pyr-HC is an analog of the commercially available fluorescent probe Pyr-AMC, which is composed of the 7-hydroxy-coumarin dye, conjugated to the enzymatic substrate via a self-immolative spacer (Figure 2A).

**Figure 2:**
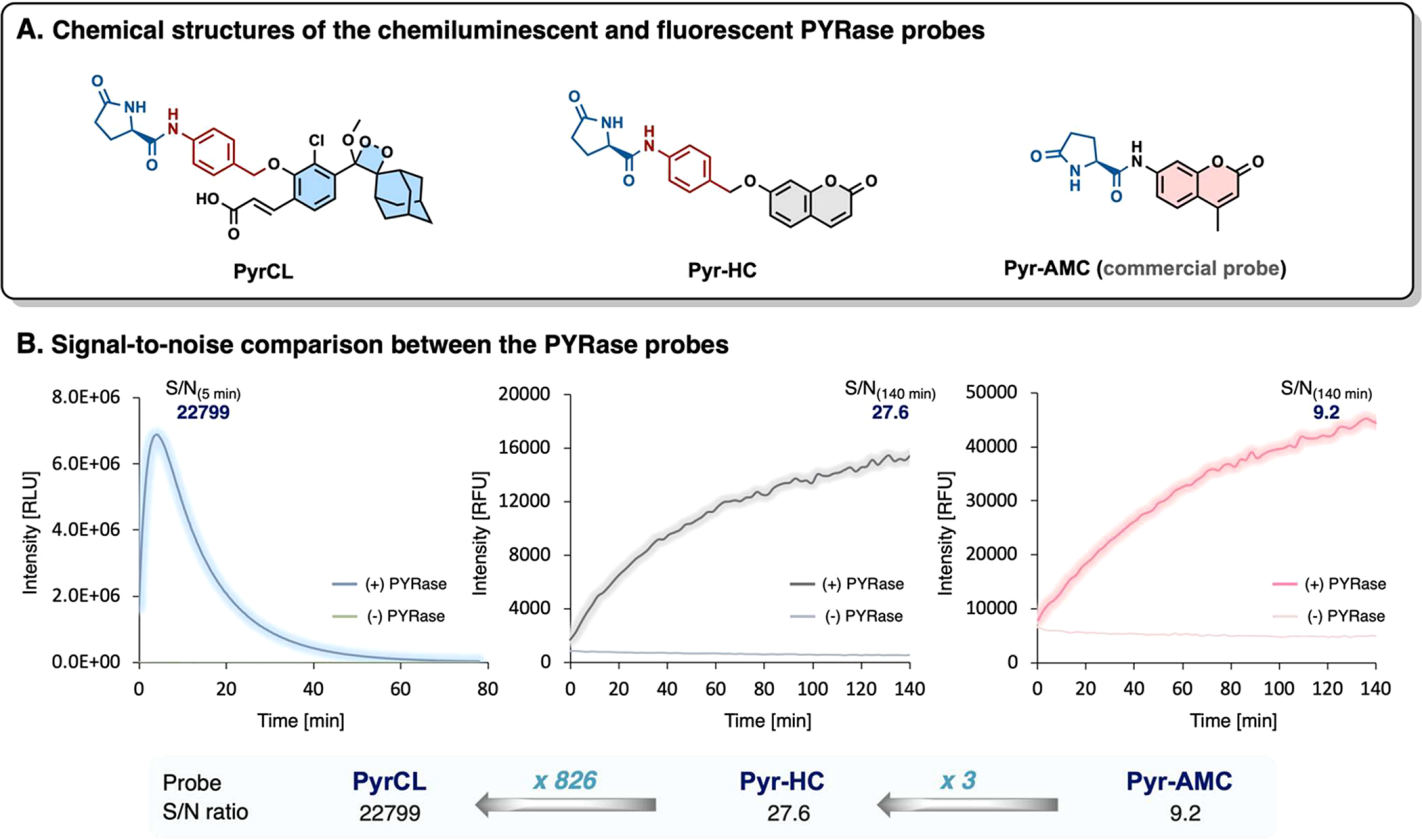
(A) Chemical structure of the chemiluminescent probe PyrCL (left), the fluorescent analog Pyr-HC (middle) that utilizes a similar self-immolative spacer as PyrCL, and the commercial fluorescent probe for PYRase Pyr-AMC (right). (B) Chemiluminescent and fluorescent kinetic profiles and S/N ratios of the three probes in the presence of PYRase.

The S/N values of the three probes were determined in the presence and absence of PYRase. Probe PyrCL exhibited maximal signal intensity after 5 min with S/N of 22799, while probes Pyr-HC and Pyr-AMC achieved S/N ratio values of 27.6-fold and 9.2-fold, with maximal signal intensity after 140 min (Figure 2B). The 826-fold and 2478-fold improvement in S/N ratio, obtained by PyrCL compared to Pyr-HC and Pyr-AMC, respectively, demonstrates the superior detection sensitivity toward PYRase activity, of the chemiluminescent probe PyrCL versus the fluorescent probes.

Next, we sought to evaluate the ability of the probe PyrCL to detect PYRase activity in Gram-positive bacteria. A schematic representation of the probe PyrCL chemiluminescent activation pathway in the presence of bacterial PYRase is shown in Figure 3A. PYRase activity was previously reported in *Streptococcus pyogenes* and *Enterococci* species.^8,29−31^ Therefore, we initially evaluated the performance of probe PyrCL in the presence of *S. pyogenes* (ATCC 14289) and *E. faecalis* (ATCC 29212). Expectedly, the incubation of probe PyrCL in the presence of *S. pyogenes* and *E. faecalis* resulted in high signal-to-noise ratios (S/N) of 878 and 1557-fold, respectively (Figure 3B).

**Figure 3:**
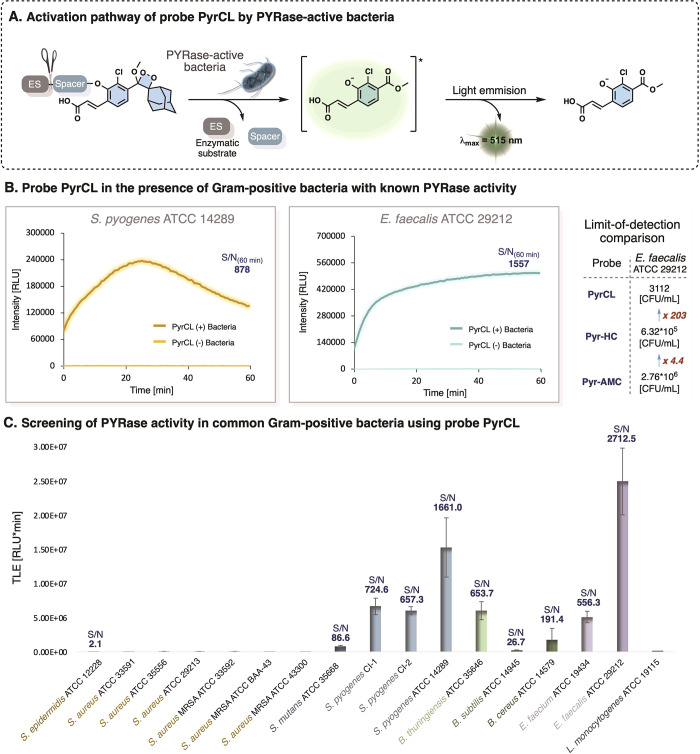
(A) Chemiluminescent activation of probe PyrCL in the presence of PYRase-active bacteria. (B) Kinetic profiles of PyrCL with PYRase-active *S. pyogenes* ATCC 14289 (left) and *E. faecalis* ATCC 29212 (middle); and a comparison between PyrCL, Pyr-HC, and Pyr-AMC limit-of-detection of *E. faecalis* ATCC 29212 (right). (C) Screening of PYRase activity in 17 strains of Gram-positive bacteria that represent the most common Gram-positive bacterial species. Error bars represent ± SD of three independent measurements.

We then conducted a comparative evaluation of the limit-of-detection (LOD) values of the chemiluminescent probe PyrCL with that of fluorescent probes Pyr-HC and Pyr-AMC, in the presence *E. faecalis* (see Figures S6–S11). Probe PyrCL exhibited an LOD value of 3.11 × 10^3^ CFU mL^–1^ while probes Pyr-HC and Pyr-AMC detected *E. faecalis* with an LOD value of 6.32 × 10^5^ CFU mL^–1^ and 2.76 × 10^6^ CFU mL^–1^, respectively (see summary in Figure 3B, right). Notably, the time required for probe PyrCL to reach the minimal LOD value was approximately 10-fold shorter than the time required for probes Pyr-HC and Pyr-AMC to reach their minimal LOD values (Figure S12). The high sensitivity exhibited by probe PyrCL toward the detection of PYRase activity prompted us to evaluate this probe’s ability to detect PYRase activity in 10 distinct Gram-positive bacterial species (Figure 3C). Probe PyrCL was incubated in the presence of *Staphylococcus epidermidis*, *Staphylococcus aureus*, *Streptococcus mutans*, *Streptococcus pyogenes*, *Bacillus thuringiensis*, *Bacillus subtilis*, *Bacillus cereus*, *Enterococcus faecium*, *Enterococcus faecalis*, and *Listeria monocytogenes* in PBS, pH 7.4, and the light emission signal was monitored over 60 min. *S*. *aureus*, *S*. *epidermidis*, and *L*. *monocytogenes* exhibited negligible PYRase activity (S/N values of 1.0–2.1). These three species served as negative controls as they do not possess PYRase activity.^8,10,31^*Bacillus* species exhibited variable levels of PYRase activity ranging from mild activity in *B. subtilis* (S/N value of 26) to high activity in *B. thuringiensis* (S/N value of 653). The *S. pyogenes* strains and *Enterococci* species exhibited high PYRase activities (S/N values of 657 to 2712-fold), which demonstrates the ability of probe PyrCL to differentiate between the *S. pyogenes* and *Enterococci* species from all other cocci strains in our tested panel. Interestingly, our chemiluminescent probe revealed a mild PYRase activity in *S. mutans* (S/N value of 86.96), which has been undetected so far.^7,8,29,31^ These observations emphasize the superior detection sensitivity of the chemiluminescent probe PyrCL compared with the other common fluorescent probes.

The ability of probe PyrCL to provide sensitive and rapid detection of PYRase activity in Gram-positive bacteria encouraged us to explore its performance in Gram-negative bacterial strains as well. PYRase activity was first identified in the genus *Pseudomonas*, while the genus *Escherichia* is reported to lack PYRase activity.^2,9^ Therefore, we initially evaluated the behavior of probe PyrCL in the presence of *Pseudomonas aeruginosa* (ATCC 47085) and *Escherichia coli* (ATCC 9637). The incubation of the probe with *P. aeruginosa* resulted in an immediate strong signal with a S/N value of 110.2 (Figure 4A). On the other hand, the incubation of the probe with *E. coli* yielded no significant response, as indicated by a negligible S/N value of 1.2. We next sought to compare the ability of probe PyrCL to detect *P. aeruginosa* with that of the fluorescent probes Pyr-AMC and Pyr-HC. The signal-to-noise ratios obtained by probe PyrCL and by the fluorescent analogs Pyr-HC and Pyr-AMC for different bacteria concentrations are shown in Figure 4B. Probe PyrCL exhibited an LOD value of 1 × 10^5^ CFU mL^–1^, which was 228-fold and 912-fold greater than the LOD values of Pyr-HC and Pyr-AMC, respectively. Next, we used probe PyrCL to screen 15 strains of Gram-negative bacteria (from seven different species of Gram-negative bacteria) for the PYRase activity. Interestingly, all four strains of the *P. aeruginosa* strains that were tested presented significantly higher PYRase activity (S/N value of 70–129) compared to the rest of the bacteria (S/N values of 1.5–5.1) in the panel (Figure 4C). This observation suggests that *P. aeruginosa* strains can be uniquely identified based on their PYRase activity by the PyrCL probe.

**Figure 4:**
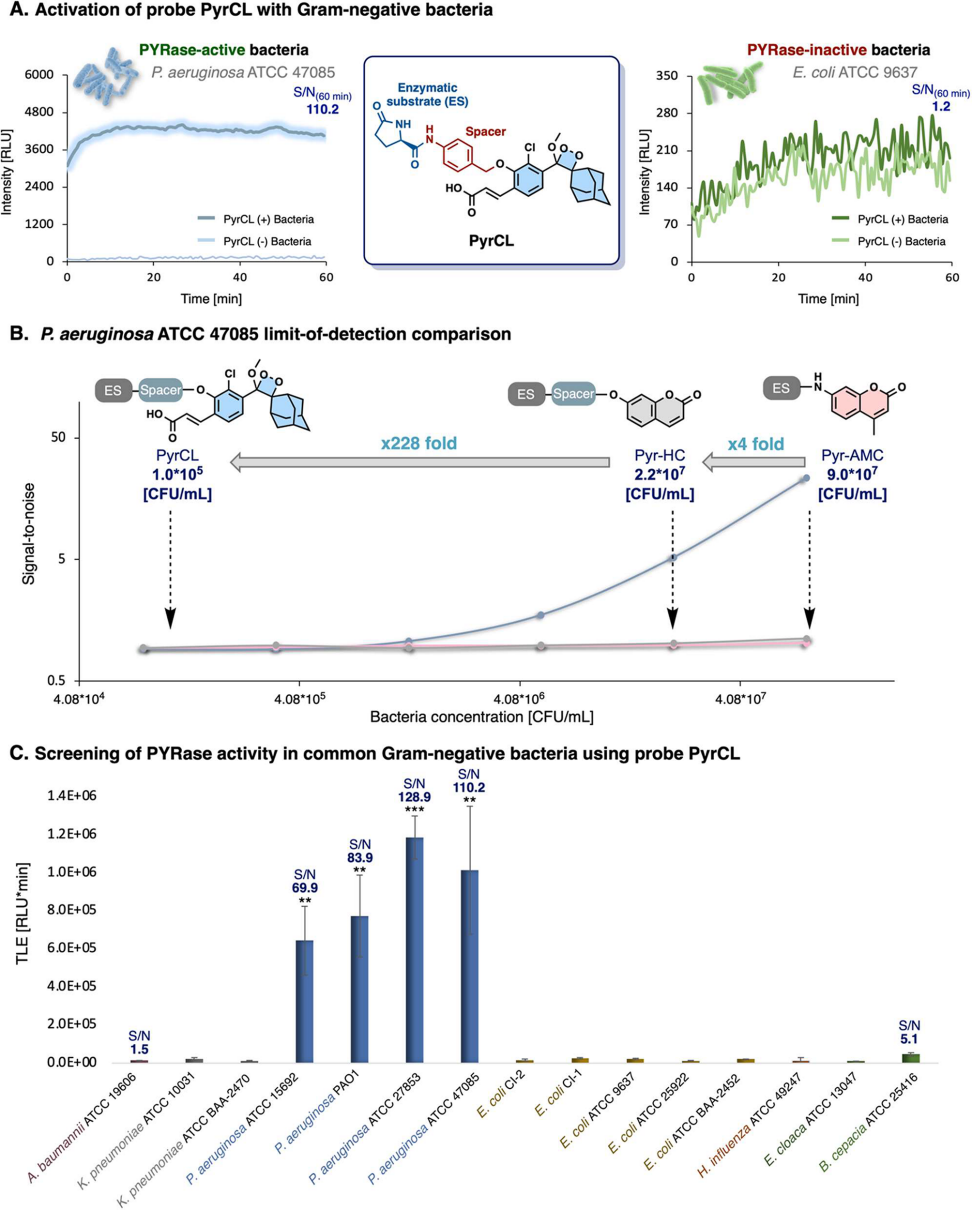
(A) Kinetic profiles of probe PyrCL with PYRase-active *P. aeruginosa* ATCC 47085 (left) and PYRase-inactive *E. coli* ATCC 9637 (right) and the chemical structure of probe PyrCL highlighting main structural motifs (middle). (B) Comparison between probes PyrCL, Pyr-HC, and Pyr-AMC limit-of-detection of *P. aeruginosa* ATCC 47085. (C) Screening of PYRase activity in 15 strains of Gram-negative bacteria that represent the most common Gram-negative bacterial species. Error bars represent ±SD of three independent measurements. Significance was determined by an unpaired student’s *t* test (*P ≤ 0.05, **P ≤ 0.01, ***P ≤ 0.001).

Probe PyrCL was recently utilized as one of 12 components in a chemiluminescent probe array that facilitates enzymatic activity profiling for general bacterial classification.^24^ Currently, many bacterial discrimination methods, relying on PYRase activity, are designed for a qualitative end-point determination.^9^ However, such an approach suffers from a fundamental drawback: bacteria with potentially high PYRase activity cannot be differentiated from those with low PYRase activity. An effective solution for this limitation is provided by our chemiluminescence probe, which enables the highly sensitive and quantitative detection of PYRase activity. The chemiluminescent screening for PYRase activity, presented in this study yields results that exemplify the potential for clinically relevant differentiation between *P*. *aeruginosa* and other Gram-negative bacteria.

The top four species isolated from clinical bacteremia (bloodstream infections) include *E. coli*, *K. pneumoniae*, *P. aeruginosa*, and *A. baumannii*.^32^ Since *P. aeruginosa* bacteremia is clinically indistinguishable from other Gram-negative bacteremia, an inappropriate initial antimicrobial therapy can lead to severe adverse outcomes.^33^ In our study, the four leading Gram-negative bacteria were evaluated for their PYRase activity. Five strains of *E. coli* including ATCC 9637, ATCC 25922, ATCC BAA-2452, and two clinical isolates; *K. pneumoniae* ATCC 10031 and ATCC BAA-2470; and *A. baumannii* ATCC 19606 were all negative for PYRase activity with S/N values ranging from 1.4 to 2.9. On the other hand, *P. aeruginosa* ATCC 15692, ATCC 27853, ATCC 47085, and the strain PAO1 all showed statistically significant increased PYRase activities with S/N values between 70 and 129. These results effectively demonstrate that the rapid detection of PYRase activity with probe PyrCL can be used to differentiate *P. aeruginosa* from the other three most prevalent Gram-negative bacteria in bacteremia. However, given the study’s restricted species and evaluated strains, a more comprehensive evaluation is needed to determine the diagnostic efficacy of probe PyrCL as a tool for identifying *P. aeruginosa*.

To date, most PYRase tests have been used routinely in clinical laboratories for differentiation between Gram-positive bacteria. It is noteworthy that in Mulczyk and Szewczuk’s screening of PYRase activity, the majority of Gram-negative bacteria were found to be PYRase-negative (1903 out of 2354 samples), including 50% of the *Pseudomonas* species that were tested.^6^ Based on the inferior sensitivity measured for fluorescence methods, it is possible that specific PYRase activities in bacteria were misclassified based on false-negative results. Therefore, the superior sensitivity of probe PyrCL may be of great diagnostic value, enabling the discovery of previously undetected PYRase activity in the bacterial domain.

In summary, we have introduced an efficient chemiluminescent probe, PyrCL, for the direct detection of PYRase activity. The probe activation mechanism is based on the hydrolytic cleavage of a pyroglutamyl substrate, followed by a rapid chemiexcitation process that results in the emission of a green photon. Probe PyrCL exhibits a significant turn-on response upon reaction with PYRase with a notably high signal-to-noise ratio (S/N = 22799 after 5 min). Comparing the chemiluminescent probe PyrCL with the fluorescent analogue probes revealed improved detection capability in terms of response time and sensitivity. Probe PyrCL was used to explore PYRase activity among 32 strains of both Gram-positive and Gram-negative bacteria from 16 distinct species. Interestingly, this screen revealed previously undetected PYRase activity in *S. mutans* and a notably high PYRase activity in *P. aeruginosa* compared to that of other Gram-negative bacteria in the evaluated panel. These findings are attributed to the superior sensitivity of our probe compared to the commercially available probes, enabling the detection of new PYRase activities in bacteria that were previously undetectable. Since the distribution of PYRase is typically characteristic of the differentiation between bacterial species, we expect that probe PyrCL, may be of high value for PYRase-based bacteria diagnosis, with relevancy in both basic research and clinical applications.

## Supporting Information Available

Experimental procedures, compound characterization data, bioassay information (PDF)
