# Supplementary material for: Hyper-Responsive Chemiluminescent Probe Reveals Distinct PYRase Activity in Pseudomonas aeruginosa
Source: Bioconjug Chem. 2024 Mar 22;35(4):472–9. doi: 10.1021/acs.bioconjchem.4c00015 (PMC11036351; doi:10.1021/acs.bioconjchem.4c00015)
Supplement: Supplementary file 1 — bc4c00015_si_001.pdf [file bc4c00015_si_001.pdf]

Supporting Information

Hyper-Responsive Chemiluminescent Probe Reveals a Distinct PYRase Activity in *Pseudomonas aeruginosa*

Rozan Tannous[a#], Omri Shelef[a#], Tal Kopp[a#], Micha Fridman[a], and Doron Shabat[a]*

[a]School of Chemistry, Raymond and Beverly Sackler Faculty of Exact Sciences, Tel-Aviv University, Tel Aviv 69978 Israel.

[#]These authors contributed equally

***Corresponding Authors:**

Doron Shabat, Email: chdoron@tauex.tau.ac.il

Table of Contents

Synthetic procedures and characterization of the PYRase probes S4

Chemiluminescent PYRase probe- PyrCL S4

Fluorescent PYRase probe- Pyr-HC.. S6

List of bacterial strains and protocols for measurements of PYRase activity in bacteria S7

List of bacterial strains S7

Protocol for limit-of-detection measurements S8

Protocol for substrate-specificity evaluation S8

Protocol for chemiluminescent measurements of PYRase activity in bacteria S8

Supplementary figures S9

NMR spectra...S18

HPLC spectra of key compounds S24

References S28

General methods

All reactions requiring anhydrous conditions were performed under an Argon atmosphere. All reactions were carried out at room temperature unless stated otherwise. Chemicals and solvents were either A.R. grade or purified by standard techniques. Thin-layer chromatography (TLC): silica gel plates Merck 60 F254: compounds were visualized by irradiation with UV light. Column chromatography (FC): silica gel Merck 60 (particle size 0.040-0.063 mm), eluent given in parentheses. Reverse-phase high-pressure liquid chromatography (RP-HPLC): C18 5u, 250x4.6mm, eluent given in parentheses. Preparative RP-HPLC: C18 5u, 250x21mm, eluent given in parentheses. ^{1}H-NMR spectra were measured using Bruker Avance operated at 400MHz. ^{13}C-NMR spectra were measured using Bruker Avance operated at 100 MHz. Chemical shifts were reported in ppm on the δ scale relative to a residual solvent ($CDCl_3$: δ = 7.26 for ^{1}H-NMR and 77.16 for ^{13}C-NMR, DMSO-d_6: δ = 2.50 for ^{1}H-NMR and 39.52 for ^{13}C-NMR). Multiplicities are reported with the following abbreviations: br, broad; s, singlet; d, doublet; t, triplet; dt, doublet of triplets; dd, doublet of doublets; ddd, doublet of doublet of doublets; qd, quartet of doublets; m, multiplet; eq, equatorial; ax, axial. Coupling constants (J) are given in Hertz. Mass spectra were measured on Waters Xevo TQD. Chemiluminescence was recorded on Molecular Devices Spectramax iD3. Fluorescence was recorded on Tecan infinite 200 Pro. All chemicals, unless otherwise stated, were obtained from commercial sources. Light irradiation for photochemical reactions: LED PAR38 lamp (19W, 3000K).

Abbreviations

ACN- Acetonitrile, **$CHCl_3$**- Chloroform, **DCM**- dichloromethane, **DMBA**- Dimethyl barbituric acid, **DMF**- N,N' – Dimethylformamide, **EEDQ**- N-Ethoxycarbonyl-2-ethoxy-1,2-dihydroquinoline, **EtOAc**- Ethyl acetate, **Hex**- Hexanes, **K_2CO_3**- Potassium carbonate, **LiOH**- Lithium hydroxide, **MB**- Methylene blue, **MeOH**- Methanol, **NH_4Cl**- ammonium chloride, **$NaHCO_3$**- Sodium bicarbonate, **$Na_2S_2O_3$**- Sodium Thiosulfate, **Na_2SO_4**- Sodium Sulfate, **$NaBH_4$**- Sodium borohydride, **NaI**- Sodium Iodide, **THF**- Tetrahydrofuran, **TMS-Cl** - Trimethylsilyl chloride.

Synthetic procedures and characterization of the PYRase probes

Chemiluminescent PYRase probe- PyrCL

PyrCL was synthesized according to our recently reported procedure.[1]

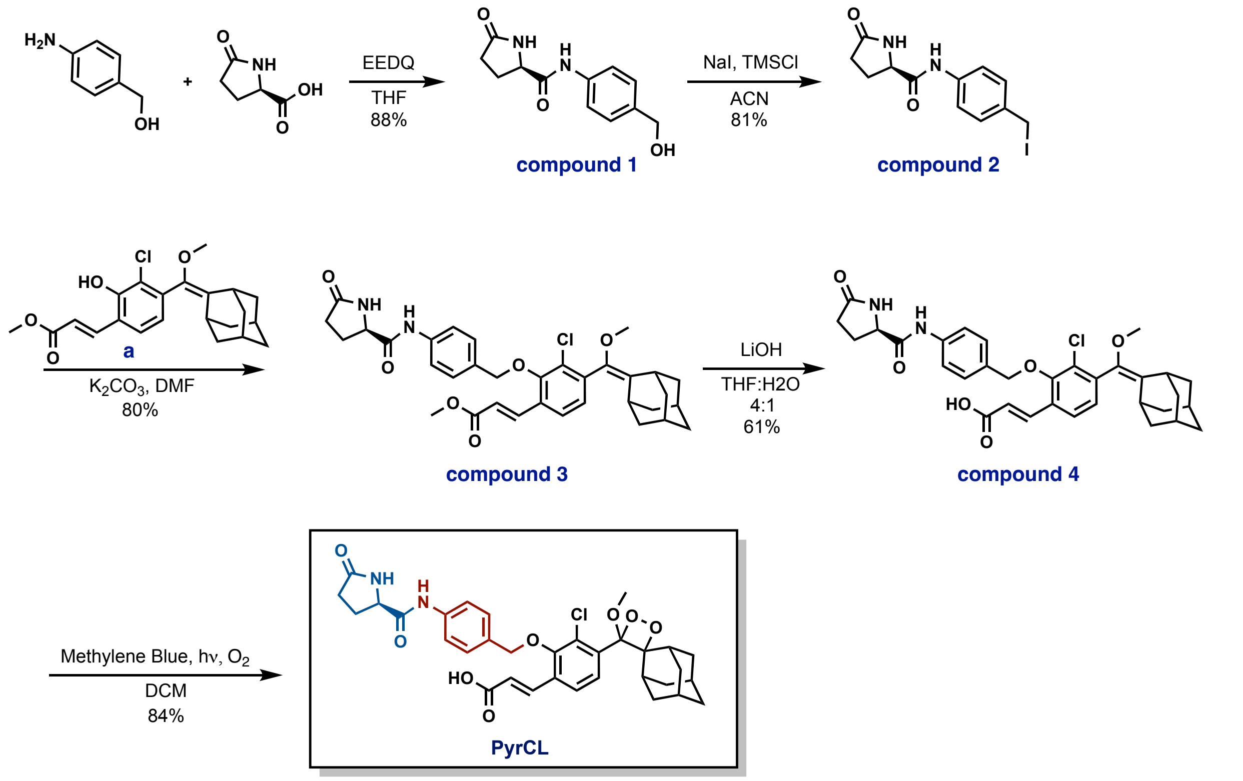

Scheme S1. Probe PyrCL synthesis

Compound 1

4-amino benzyl alcohol (262 mg, 2.13 mmol, 1.1 eq.) and L-Pyroglutamic Acid (250 mg, 1.93 mmol, 1 eq.) were dissolved in 3 mL of THF followed by the addition of EEDQ (957 mg, 3.87 mmol, 2 eq.). The reaction mixture was stirred at room temperature and monitored by TLC (EtOAc: Hex mixture). Upon completion, the reaction mixture was filtered and washed three times with Et_2O, to afford compound **1** (400 mg, 88% yield) as a white solid.
^{1}H NMR (400 MHz, DMSO) δ 10.00 (s, 1H), 7.88 (s, 1H), 7.56 (d, J = 8.5 Hz, 2H), 7.25 (d, J = 8.6 Hz, 2H), 5.10 (t, J = 5.7 Hz, 1H), 4.43 (d, J = 5.6 Hz, 2H), 4.18 (dd, J = 8.5, 4.4 Hz, 1H), 2.41 – 1.91 (m, 4H).
^{13}C NMR (100 MHz, DMSO) δ 177.91, 171.62, 138.13, 137.86, 127.40, 119.57, 63.03, 56.83, 29.72, 25.78.
MS (ES+): m/z calc. for $C_{12}H_{14}N_2O_3$:234.10; found: 257.2 $[M+Na]^+$.

Compound 2

Compound **1** (400 mg, 1.71 mmol, 1 eq.) was dissolved in 4 mL of ACN and cooled to 0 °C. Sodium Iodide (764 mg, 5.13 mmol, 3 eq.) was added followed by the rapid addition of TMS-Cl (647 µl, 5.13 mmol, 3 eq.). The reaction was allowed to warm up to room temperature and monitored by TLC (EtOAc: Hex mixture). Upon completion, the reaction mixture was diluted with EtOAc, and washed with saturated $Na_2S_2O_3$ followed by brine. The organic layer

was separated, dried over Na_2SO_4, filtered and the solvent was evaporated under reduced pressure. The crude product was further purified by column chromatography (EtOAc: Hex mixture) to afford compound **2** in the form of a yellow solid (476 mg, 1.38 mmol, 81%).

^{1}H NMR (400 MHz, CDCl3) δ 7.52 (dd, J = 8.5, 3.7 Hz, 2H), 7.38 – 7.30 (m, 2H), 4.45 (d, J = 3.2 Hz, 2H), 4.27 (ddd, J = 8.8, 6.8, 4.8 Hz, 1H), 3.44 – 3.34 (m, 1H), 2.65 – 2.29 (m, 4H).

^{13}C NMR (101 MHz, DMSO) δ 177.91, 172.26, 141.24, 132.92, 129.96, 120.33, 118.30, 57.19, 56.86, 29.66, 25.83.

Compound 3

Phenol enol ether **b**[2] (796 mg, 2.05 mmol, 1.2 eq.) and K_2CO_3 (354 mg, 2.56 mmol, 1.5 eq.) were dissolved in DMF (5 mL). The solution was stirred for 5 minutes, then compound **2** was added. The reaction mixture was stirred at room temperature and monitored by TLC (EtOAc:Hex mixture). Upon completion, the reaction mixture was diluted with EtOAc (100 mL) and washed with 1M HCl (50 mL) and brine (50 mL). The organic layer was separated, dried over Na_2SO_4, and evaporated under reduced pressure. The crude product was purified by column chromatography on silica gel (EtOAc: Hex mixture) to afford compound **3** (827 mg, 80% yield) as a white solid.

^{1}H NMR (400 MHz, CDCl3) δ 8.89 (s, 1H), 7.88 (d, J = 16.2 Hz, 1H), 7.64 (d, J = 8.4 Hz, 2H), 7.49 (s, 1H), 7.45 – 7.38 (m, J = 8.2, 4.1 Hz, 3H), 7.08 (d, J = 8.0 Hz, 1H), 6.42 (d, J = 16.2 Hz, 1H), 4.95 (d, J = 3.7 Hz, 2H), 4.32 – 4.26 (m, J = 8.4, 4.9 Hz, 1H), 3.79 (s, 3H), 3.32 (s, 3H), 3.28 (s, 1H), 2.60 – 2.25 (m, 4H), 2.08 (s, 1H), 2.00 – 1.65 (m, 12H).

^{13}C NMR (100 MHz, $CDCl_3$) δ 179.64, 170.52, 167.23, 153.68, 139.41, 138.93, 138.23, 137.87, 132.48, 132.28, 129.81, 129.67, 127.82, 125.09, 120.09, 119.81, 75.72, 57.88, 57.27, 51.85, 39.19, 39.04, 38.62, 37.05, 32.94, 29.70, 29.48, 28.35, 28.20, 25.80.

MS (ES+): m/z calc. for $C_{34}H_{37}ClN_2O_6$: 604.23; found:627.6 $[M+Na]^+$

Compound 4

Compound **3** (100 mg, 0.16 mmol, 1 eq.) and LiOH (39 mg, 1.6 mmol, 10 eq.) were dissolved in 1 mL solution of THF: H_2O mixture (4:1, respectively). The reaction mixture was stirred at room temperature and monitored by RP-HPLC. Upon completion, the solvent was concentrated under reduced pressure and the product was purified by preparative RP-HPLC (mobile phase: acetonitrile in H_2O containing 0.1% TFA; gradient from 70 to 100%; flow rate: 20 mL/min). Compound **4** was obtained as a white solid (59 mg, 61% yield).

^{1}H NMR (400 MHz, $CDCl_3$) δ 7.69 (d, J = 16.1 Hz, 1H), 7.55 (d, J = 8.4 Hz, 2H), 7.37 (d, J = 8.0 Hz, 1H), 7.31 (d, J = 8.4 Hz, 2H), 7.05 (d, J = 8.0 Hz, 1H), 6.20 (d, J = 16.1 Hz, 1H), 4.96 (s, 2H), 4.33 – 4.21 (m, 1H), 3.31 (s, 3H), 3.25 (s, 1H), 2.57 – 2.24 (m, 4H), 2.06 (s, 1H), 1.98 – 1.62 (m, 12H).

^{13}C NMR (100 MHz, $CDCl_3$) δ 170.77, 168.87, 153.41, 139.34, 138.02, 137.89, 132.64, 132.04, 130.03, 129.66, 127.81, 124.89, 120.08, 119.80, 76.00, 57.61, 57.25, 39.13, 38.99, 38.60, 36.99, 32.94, 29.70, 29.44, 28.30, 28.15, 25.39.

MS (ES+): m/z calc. for $C_{33}H_{35}ClN_2O_6$: 590.22; found:613.5 $[M+Na]^+$

Probe PyrCL

Compound **4** (10 mg, 0.02 mmol) and a catalytic amount of methylene blue (~1 mg) were dissolved in 10 mL of DCM. Oxygen was bubbled through the solution while irradiating with yellow light. The reaction was monitored by RP-HPLC. Upon completion, the solvent was concentrated under reduced pressure, and the product was purified by preparative RP-HPLC (mobile phase: acetonitrile in H_2O containing 0.1% TFA; gradient from 70 to 100%; flow rate: 20 mL/min). **Probe PyrCL** was obtained as a white solid (8.8 mg, 84% yield).

^{1}H NMR (400 MHz, $CDCl_3$) δ 8.81 (s, 1H), 7.97 – 7.89 (m, 1H), 7.66 (dd, J = 26.2, 16.1 Hz, 1H), 7.56 (t, J = 6.5 Hz, 2H), 7.19 (dd, J = 12.2, 8.4 Hz, 2H), 6.27 (dd, J = 21.5, 16.1 Hz, 1H), 5.06 (dd, J = 31.5, 11.4 Hz, 1H), 4.85 (dd, J = 39.4, 11.3 Hz, 1H), 4.42 (s, 1H), 3.28 (d, J = 4.8 Hz, 3H), 3.06 (s, 1H), 2.67 – 2.26 (m, 4H), 2.04 (s, 1H), 1.94 – 1.24 (m, 12H).
^{13}C NMR (100 MHz, $CDCl_3$) δ 181.34, 181.16, 171.03, 169.74, 139.82, 139.73, 138.04, 135.44, 132.11, 131.91, 131.61, 130.36, 130.28, 128.93, 127.69, 124.83, 119.98, 111.75, 96.37, 58.48, 49.77, 36.58, 33.92, 33.62, 32.66, 32.23, 31.56, 29.57, 26.15, 25.86, 25.46.
MS (ES+): m/z calc. for $C_{33}H_{35}ClN_2O_8$: 622.21; found:645.5 $[M+Na]^+$.

Fluorescent PYRase probe- Pyr-HC

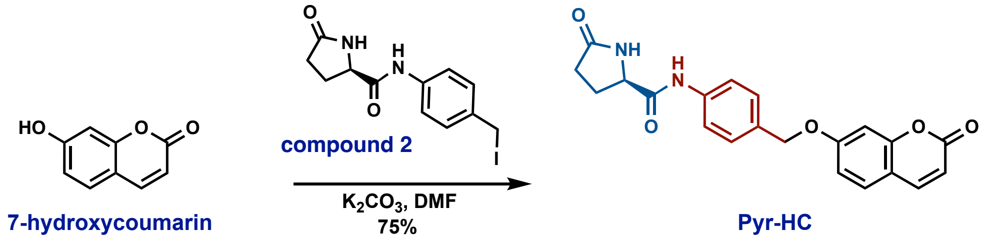

Scheme S2. Probe Pyr-HC synthesis.

Pyr-HC

7-hydroxycoumarin (12 mg, 0.07 mmol, 1.2 eq.) and K_2CO_3 (12 mg, 0.09 mmol, 1.5 eq.) were dissolved in DMF (1 mL). The solution was stirred for 5 minutes followed by the addition of compound **2** (21 mg, 0.06 mmol, 1 eq.). The reaction mixture was stirred at room temperature and monitored by TLC (EtOAc/Hex mixture). Upon completion, the reaction mixture was diluted with EtOAc (100 mL) and washed with 1M HCl (50 mL) and brine (50 mL). The organic layer was separated, dried over Na_2SO_4, and evaporated under reduced pressure. The crude product was purified by preparative RP-HPLC (mobile phase: acetonitrile in H_2O containing 0.1% TFA; gradient from 30 to 100%; flow rate: 20 mL/min). Probe **Pyr-HC** was obtained as a white solid (17 mg, 75% yield).
^{1}H NMR (400 MHz, DMSO) δ 10.10 (s, 1H), 7.95 (d, *J* = 9.5 Hz, 1H), 7.88 (s, 1H), 7.63 (d, *J* = 8.5 Hz, 2H), 7.60 (d, *J* = 8.7 Hz, 1H), 7.41 (d, *J* = 8.5 Hz, 2H), 7.04 (d, *J* = 2.3 Hz, 1H), 6.98 (dd, *J* = 8.6, 2.4 Hz, 1H), 6.26 (d, *J* = 9.5 Hz, 1H), 5.13 (s, 2H), 4.18 (dd, *J* = 8.5, 4.3 Hz, 1H), 2.38 – 2.26 (m, 1H), 2.26 – 2.06 (m, 2H), 2.02 – 1.92 (m, 1H).
^{13}C NMR (100 MHz, DMSO) δ 178.07, 171.99, 162.05, 160.86, 155.91, 144.88, 139.31, 131.75, 130.08, 129.31, 119.93, 113.62, 113.17, 113.07, 102.21, 70.27, 56.99, 29.82, 25.92.
MS (ES+): m/z calc. for $C_{21}H_{18}N_2O_5$: 378.12; found:401.4 $[M+Na]^+$

List of bacterial strains and protocols for measurements of PYRase activity in bacteria

List of bacterial strains

	#	Species	Strain name	Source	Growth condition
Gram-positive bacteria					
1	1.	*Streptococcus pyogenes*	14289	ATCC	BHI, 37°C
	2.	*Streptococcus pyogenes*	CI-1	Clinical isolate	BHI, 37°C
	3.	*Streptococcus pyogenes*	CI-2	Clinical isolate	BHI, 37°C
2	4.	*Streptococcus mutans*	35668	ATCC	BHI, 37°C
3	5.	*Staphylococcus aureus*	35556	ATCC	LB, 37°C
	6.	*Staphylococcus aureus*	29213	ATCC	LB, 37°C
	7.	*Staphylococcus aureus* MRSA	33591	ATCC	BHI, 37°C
	8.	*Staphylococcus aureus* MRSA	33592	ATCC	BHI, 37°C
	9.	*Staphylococcus aureus* MRSA	BAA-43	ATCC	BHI, 37°C
	10.	*Staphylococcus aureus* MRSA	43300	ATCC	BHI, 37°C
4	11.	*Staphylococcus epidermidis*	12228	ATCC	LB, 37°C
5	12.	*Bacillus cereus*	14579	ATCC	LB, 30°C
6	13.	*Bacillus subtilis*	14945	ATCC	LB, 30°C
7	14.	*Bacillus thuringiensis*	35646	ATCC	LB, 30°C
8	15.	*Enterococcus faecalis*	29212	ATCC	LB, 37°C
9	16.	*Enterococcus faecium*	19434	ATCC	LB, 37°C
10	17.	*Listeria monocytogenes*	19115	ATCC	BHI, 37°C
Gram-negative bacteria					
11	18.	*Escherichia coli*	25922	ATCC	LB, 37°C
	19.	*Escherichia coli*	9637	ATCC	LB, 37°C
	20.	*Escherichia coli*	CI-1	Daniel Kahne group	LB, 37°C
	21.	*Escherichia coli*	CI-2	Clinical isolate	LB, 37°C
	22.	*Escherichia coli*	BAA-2452	ATCC	LB, 37°C
12	23.	*Pseudomonas aeruginosa*	47085	ATCC	LB, 37°C
	24.	*Pseudomonas aeruginosa*	15692	ATCC	LB, 37°C
	25.	*Pseudomonas aeruginosa*	PAO1	-	LB, 37°C
	26.	*Pseudomonas aeruginosa*	27853	ATCC	LB, 37°C
13	27.	*Klebsiella pneumoniae*	BAA-2470	ATCC	LB, 37°C
	28.	*Klebsiella pneumoniae*	10031	ATCC	LB, 37°C
14	29.	*Acinetobacter baumannii*	19606	ATCC	LB, 37°C
15	30.	*Haemophilus influenza*	49247	ATCC	*sBHI, 37°C
16	31.	*Burkholderia cepacia*	25416	ATCC	LB, 30°C
17	32.	*Enterobacter cloaca*	13047	ATCC	LB, 30°C

* Supplemented BHI with hemin and nicotinamide adenine dinucleotide.

Table S1. Bacteria strains.

Protocol for limit-of-detection measurements

Enterococcus faecalis ATCC 29212 and *Pseudomonas aeruginosa* ATCC 47085 were cultured in Lysogeny broth at 37°C for 18 hours under aerobic conditions. The initial culture was rinsed with PBS (centrifuged at 5000 rpm, 10 minutes), and the resulting bacterial pellet was resuspended in 4 mL of PBS to facilitate a 1:4 dilution experiment. For the subsequent procedure, a 96-well plate was utilized, with each well initially loaded with 50μL of the pyro-glutamyl aminopeptidase chemiluminescent probe (PyrCL) [20μM, 0.2% DMSO], pyro-glutamyl aminopeptidase fluorescent probe (Pyr-HC) [100μM, 0.2% DMSO], or pyro-glutamyl aminopeptidase commercial fluorescent probe (Pyr-AMC) [100μM, 0.2% DMSO]. Subsequently, 50μL of bacterial aliquot was introduced into each well, marking the commencement of the 1:4 dilution experiment, which was initiated with an OD_{600} of 0.4. The ensuing chemiluminescence signal was monitored over 4hours of incubation at 37°C using a Molecular Devices Spectramax i3x. Simultaneously, the fluorescence signal was recorded during the same timeframe at the same temperature using a Tecan Infinite 200 Pro.

Enterococcus faecalis ATCC 29212 and *Pseudomonas aeruginosa* ATCC 47085 OD_{600} values were converted to CFU/mL according to reported ratios.[3, 4]

Protocol for substrate-specificity evaluation

The specificity of the pyro-glutamyl aminopeptidase chemiluminescent probe (PyrCL) was assessed in the presence of ten commercially available recombinant enzymes. The chemiluminescent intensity the probe [10 μM in PBS (pH 7.4), 0.1% DMSO, 37°C] was measured in conjunction with the following commercially available recombinant enzymes: β-glucosidase (from almonds) [10 U/mL], β-glucuronidase (from *E. coli*) [1 U/mL], β-galactosidase (from *E. coli*) [1 U/mL], pyroglutamyl-peptidase I (from *E. coli*) [0.05 μg/mL], alkaline phosphatase (from bovine intestinal mucosa) [1 U/mL], aminopeptidase-M (from Porcine Kidney) [1 U/mL], nitroreductase (from *E. coli*) [1 μg/mL, 100μM NADH], NQO1 (from *E. coli*) [0.8 μg/mL, 100μM NADH], β-lactamase (from *E. coli*) [2 U/mL], penicillin G-amidase (from *E. coli)* [1 U/mL].

Protocol for chemiluminescent measurements of PYRase activity in bacteria

All bacterial strains were cultured in LB/BHI at either 37°C or 30°C for 18 hours under aerobic conditions. Subsequently, the initial culture was subjected to a PBS wash (centrifuged at 5000 rpm, 10 minutes), and the bacterial pellet obtained was reconstituted in 4 mL of PBS, aiming for an OD_{600} of 0.8. Following this, a 96-well plate was utilized, and each well was pre-loaded with 50 μL of the chemiluminescent probe (PyrCL) [20 μM, 0.2% DMSO]. Next, 50 μL of bacterial aliquot was introduced into each well, bringing the final OD_{600} to 0.4. The resultant chemiluminescence signal was monitored using a Molecular Devices Spectramax iD3 over 1 hour of incubation at 37°C.

Supplementary figures

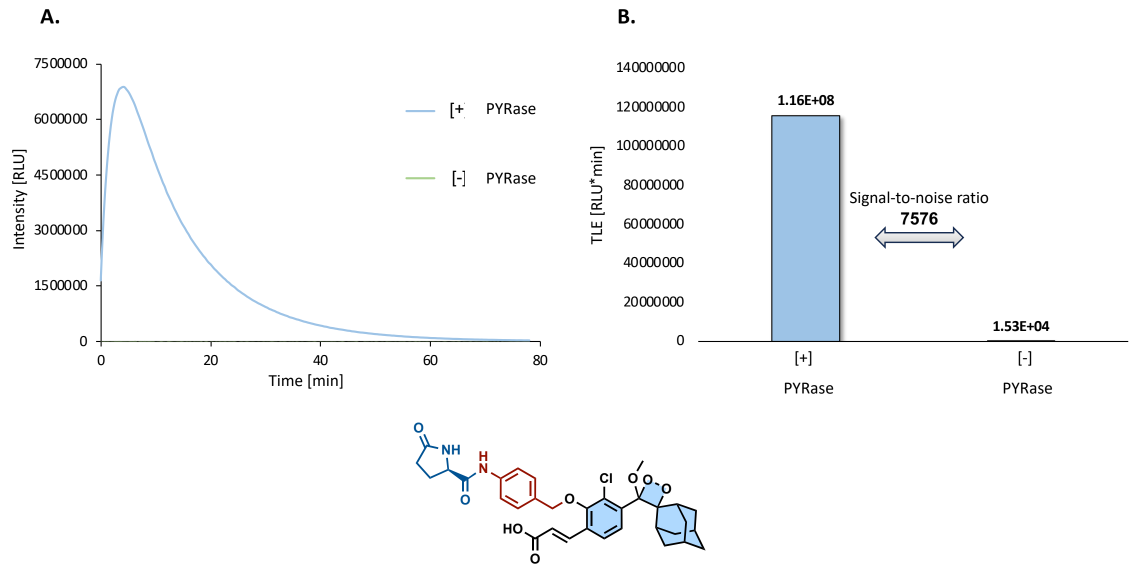

PyrCL

Figure S1. Chemiluminescent kinetic profile (**A.**), and total light emission (**B.**) of the chemiluminescent pyro-glutamyl aminopeptidase probe (PyrCL) [10 μM] in the presence and absence of pyroglutamyl-peptidase I [0.5 μg/mL] in PBS (pH 7.4), 0.1% DMSO, 37°C.

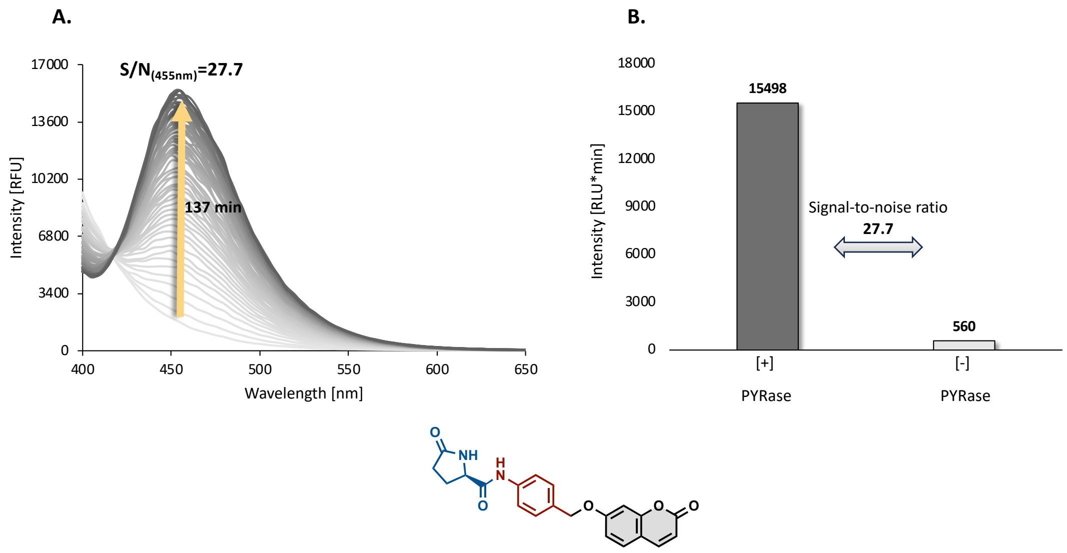

Pyr-HC

Figure S2. Fluorescent emission spectra during time (**A.**), and light intensity (**B.**) of the fluorescent pyro-glutamyl aminopeptidase probe (Pyr-HC) [50 μM] in the presence and absence of pyroglutamyl-peptidase I [0.5 μg/mL] in PBS (pH 7.4), 0.1% DMSO, 37°C. $\lambda_{ex} = 350nm$.

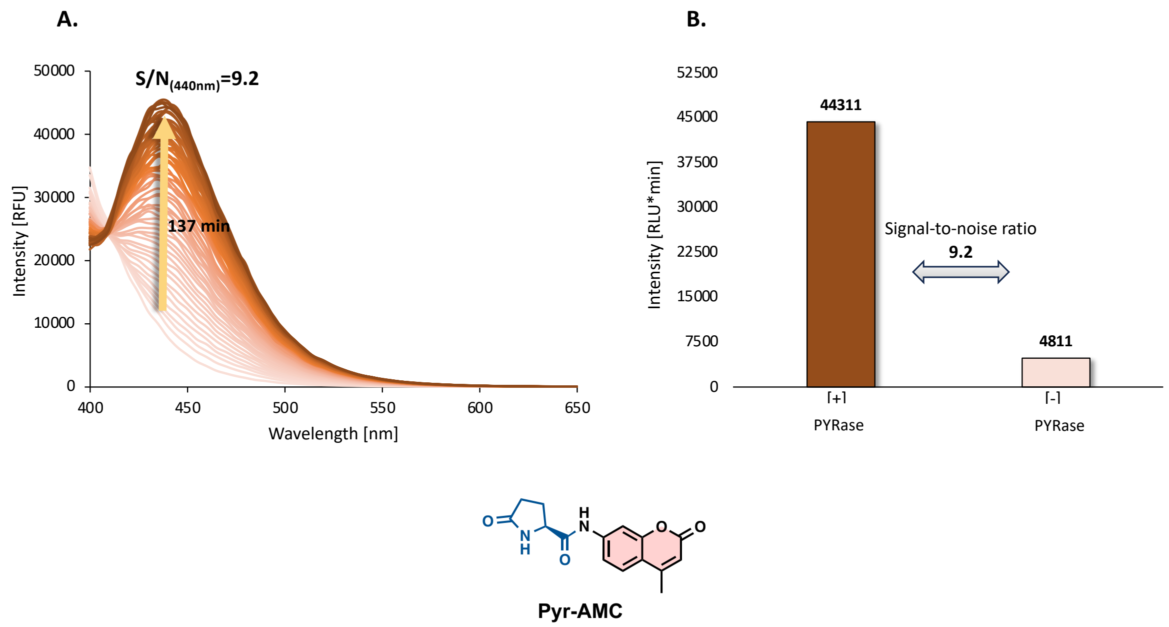

Figure S3. Fluorescent emission spectra during time (**A.**), and light intensity (**B.**) of the fluorescent pyro-glutamyl aminopeptidase commercial probe (Pyr-AMC) [50 µM] in the presence and absence of pyroglutamyl-peptidase I [0.5 µg/mL] in PBS (pH 7.4), 0.1% DMSO, 37°C. $\lambda_{ex} = 350nm$.

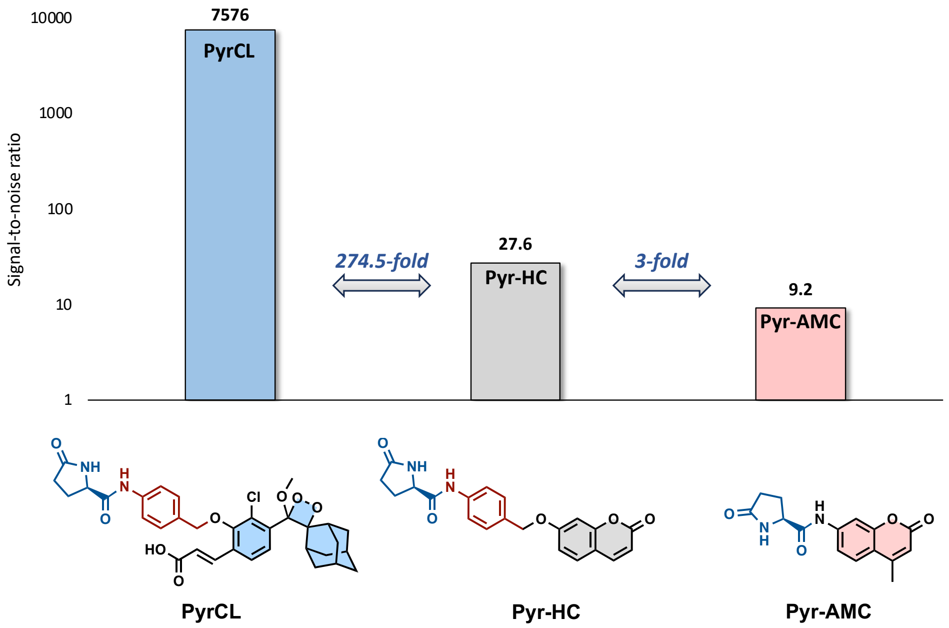

Figure S4. Signal-to-noise comparison between the pyro-glutamyl aminopeptidase chemiluminescent probe (PyrCL) [10µM] and the pyro-glutamyl aminopeptidase fluorescent probes (Pyr-HC and Pyr-AMC) [50µM] in the presence and absence of pyroglutamyl-peptidase I [0.5 µg/mL] in PBS (pH 7.4), 0.1% DMSO, 37°C.

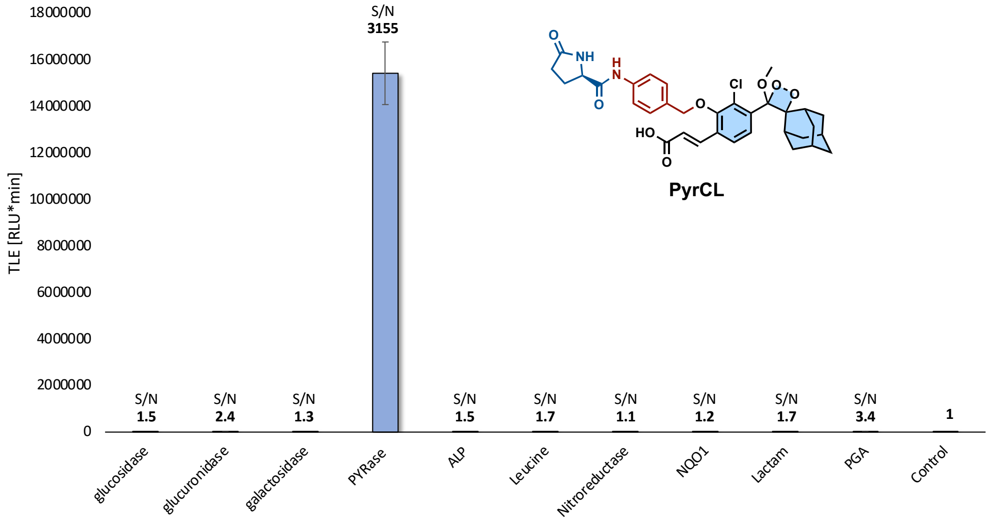

Figure S5. The selectivity of the chemiluminescent pyro-glutamyl aminopeptidase probe (PyrCL) [10 µM in PBS (7.4 pH), 0.1% DMSO, 37°C] was evaluated in the presence of 10 commercially available recombinant enzymes. The chemiluminescent intensity was measured in the presence of each of the following commercially available recombinant enzymes: β-glucosidase (from almonds) [10 U/mL], β-glucuronidase (from *E. coli*) [1 U/mL], β-galactosidase (from *E. coli*) [1 U/mL], pyroglutamyl-peptidase I (from *E. coli*) [0.05 µg/mL], alkaline phosphatase (from bovine intestinal mucosa) [1 U/mL], aminopeptidase-M (from Porcine Kidney) [1 U/mL], nitroreductase (from *E. coli*)[1 µg/mL, 100µM NADH], NQO1 (from *E. coli*) [0.8 µg/mL, 100µM NADH], β-lactamase (from *E. coli*) [2 U/mL], Penicillin-G amidase (from *E. coli*) [1 U/mL]

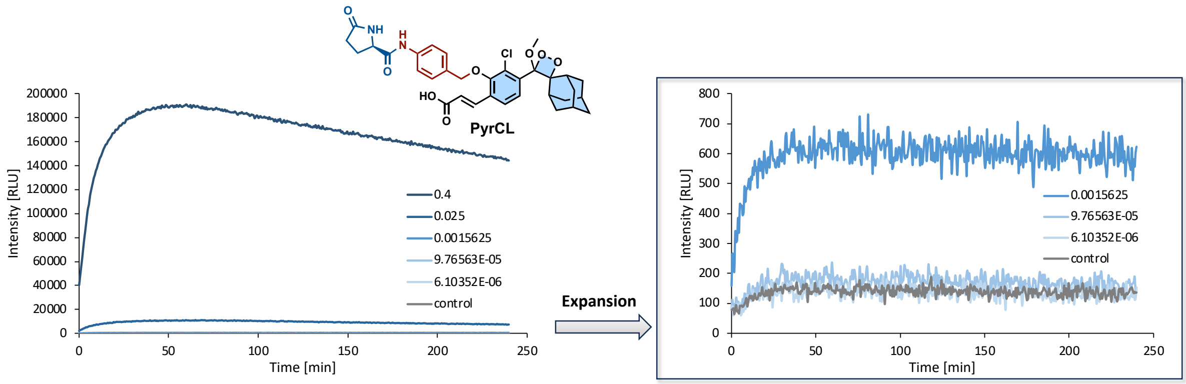

Figure S6. Chemiluminescent kinetic profiles during 4 hours of PyrCL probe [10 µM] with various bacterial optical density of *E. faecalis* ATCC 29212 [OD_{600} 0.4 - 6.10*10^{-6}] in PBS 7.4, 0.1% DMSO, 37°C.

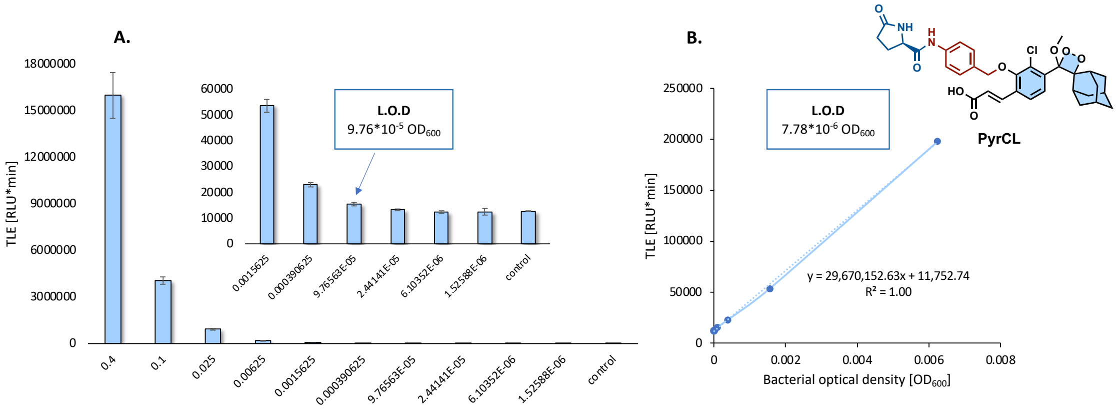

Figure S7. Total light emitted **(A.)** and linear calibration curve **(B.)** after 90 min of PyrCL probe [10 µM] with various bacterial optical densities of *E. faecalis* ATCC 29212 [OD_{600} 0.4 – $1.52*10^{-6}$] in PBS 7.4, 0.1% DMSO, 37°C. The limit of detection (L.O.D) was determined using two methods: the blank + 3SD (standard deviation) method (left), and secondly, by a linear calibration curve. For the latter, the limit of detection is defined as 3 times the standard deviation of the blank divided by the slope of the linear calibration curve (L.O.D = 3σ/k) (right). Since the second method (L.O.D = 3σ/k) is more commonly used, the results of this L.O.D comparison are presented in the manuscript.

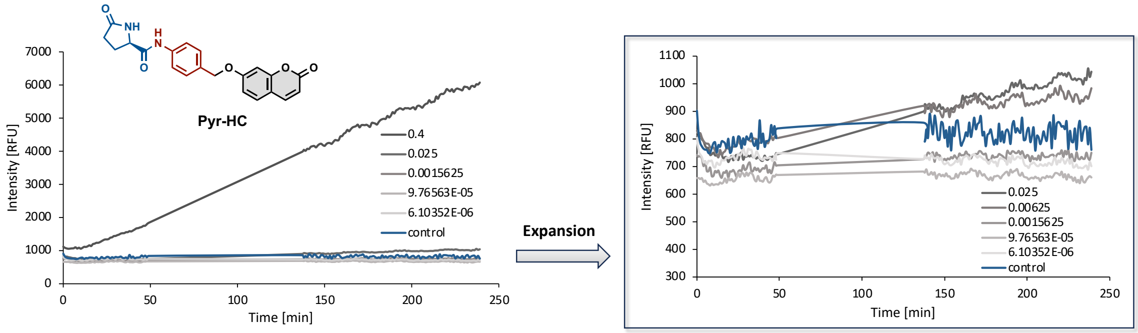

Figure S8. Fluorescent kinetic profiles during 4 hours of Pyr-HC probe [50 µM] with various bacterial optical densities of *E. faecalis* ATCC 29212 [OD_{600} 0.4 - $6.10*10^{-6}$] in PBS 7.4, 0.1% DMSO, 37°C. $\lambda_{ex} = 350nm$.

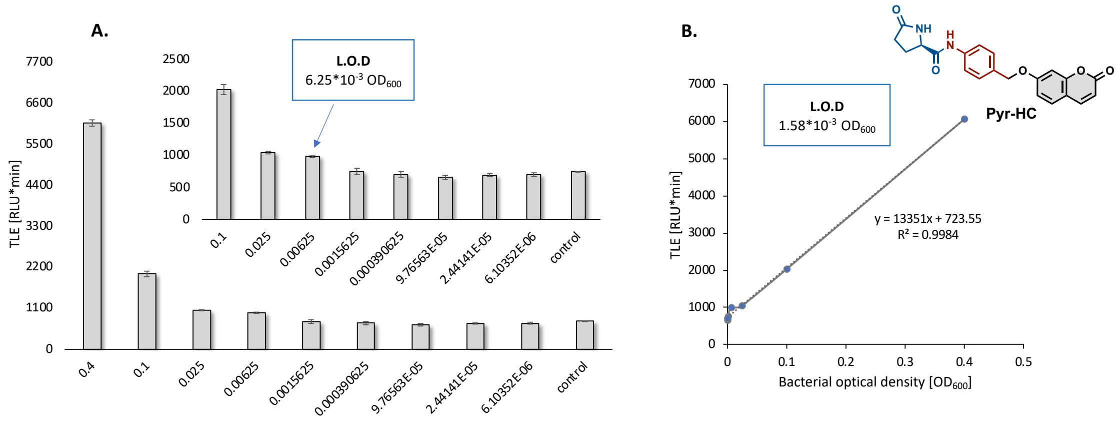

Figure S9. Light intensity **(A.)** and linear calibration curve **(B.)** after 240 min of Pyr-HC probe [50 µM] with various bacterial optical densities of *E. faecalis* ATCC 29212 [OD_{600} 0.4 - $6.10*10^{-6}$] in PBS 7.4, 0.1% DMSO, 37°C.

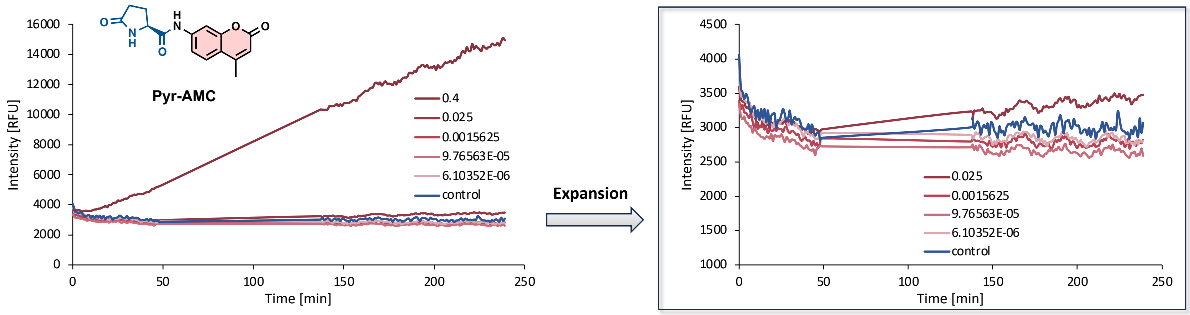

Figure S10. Fluorescent kinetic profiles during 4 hours of Pyr-AMC commercial probe [50 µM] with various bacterial optical densities of *E. faecalis* ATCC 29212 [OD_{600} 0.4 - 6.10*10^{-6}] in PBS 7.4, 0.1% DMSO, 37°C. $\lambda_{ex} = 350nm$.

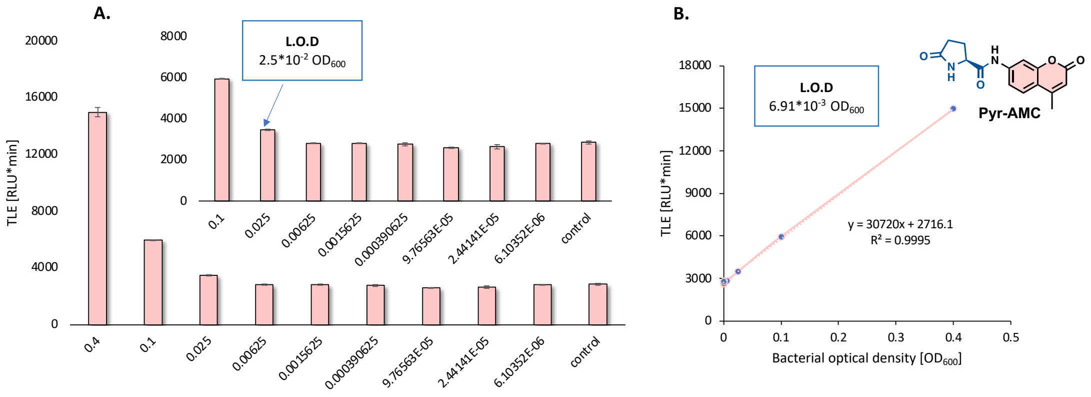

Figure S11. Light intensity **(A.)** and linear calibration curve **(B.)** after 240 min of Pyr-AMC probe [50 µM] with various bacterial optical densities of *E. faecalis* ATCC 29212 [OD_{600} 0.4 - 6.10*10^{-6}] in PBS 7.4, 0.1% DMSO, 37°C.

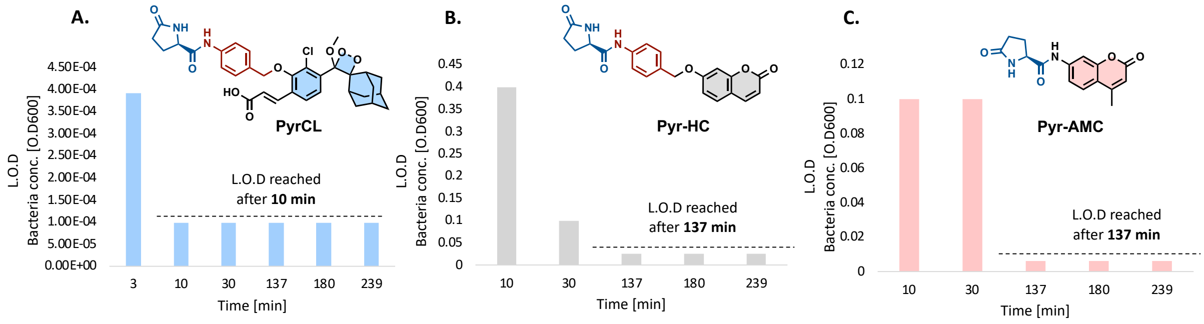

Figure S12. Limit-of-detection concentration [OD_{600}] measured in the different timeframes for PyrCL probe [10 µM], Pyr-HC and Pyr-AMC probes [50 µM] in the presence and absence of various bacterial optical densities of *E. faecalis* ATCC 29212 [OD_{600} 0.4 - 6.10*10^{-6}] in PBS 7.4, 0.1% DMSO, 37°C.

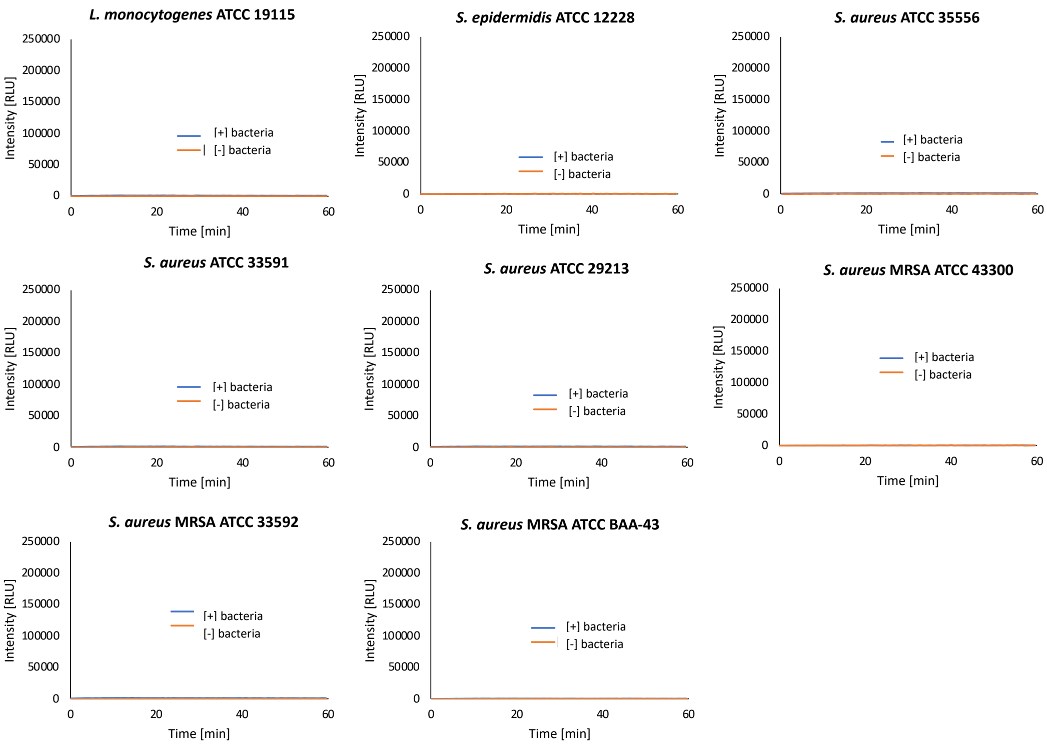

Figure S13. Chemiluminescent kinetic profiles of PyrCL probe [10µM] with eight out of the 17 strains of gram-positive bacteria, [OD_{600} 0.4], in PBS (pH 7.4), 0.1% DMSO, 37°C.

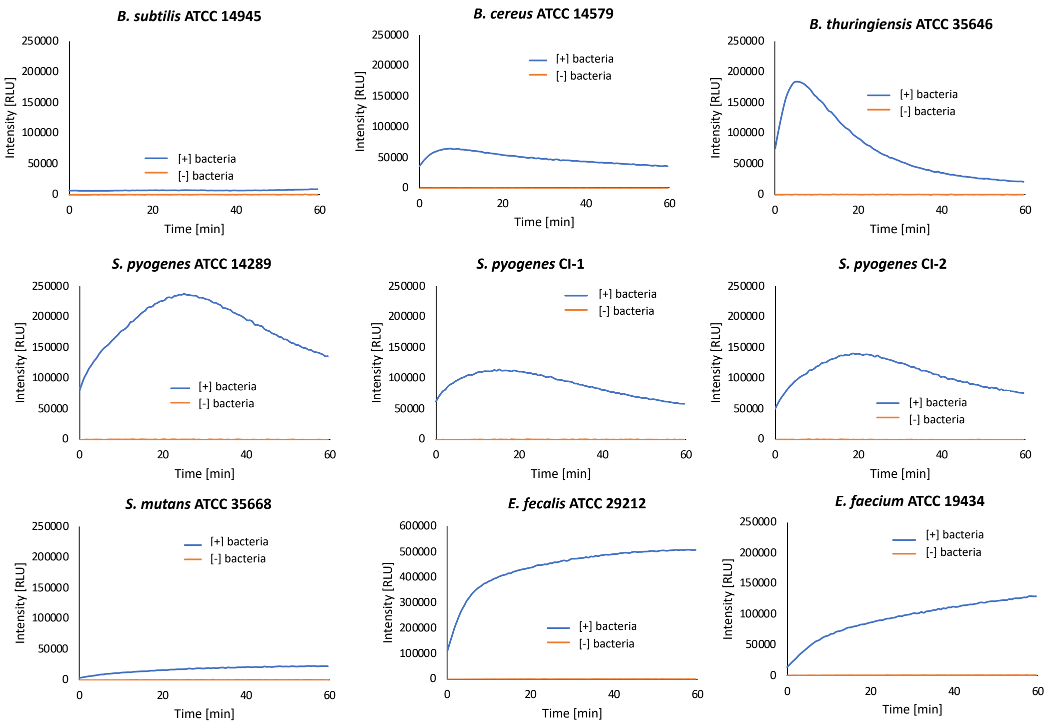

Figure S14. Chemiluminescent kinetic profiles of PyrCL probe [10µM] with nine out of the 17 strains of gram-positive bacteria, [OD_{600} 0.4], in PBS (pH 7.4), 0.1% DMSO, 37°C.

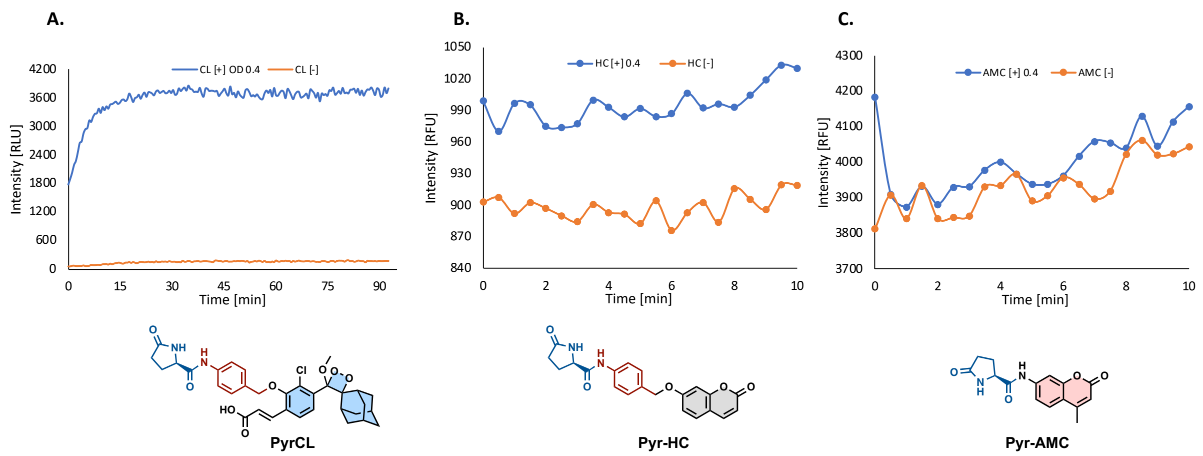

Figure S15. Chemiluminescent kinetic profile of probe PyrCL [10µM] **(A.)** and fluorescent kinetic profiles of probes Pyr-HC **(B.)** and Pyr-AMC **(C.)** [50µM] after <u>*3 hours*</u> of incubation in the presence and absence of *P. aeruginosa* ATCC 47085 [OD_{600} 0.4], in PBS (pH 7.4), 0.1% DMSO, 37°C.

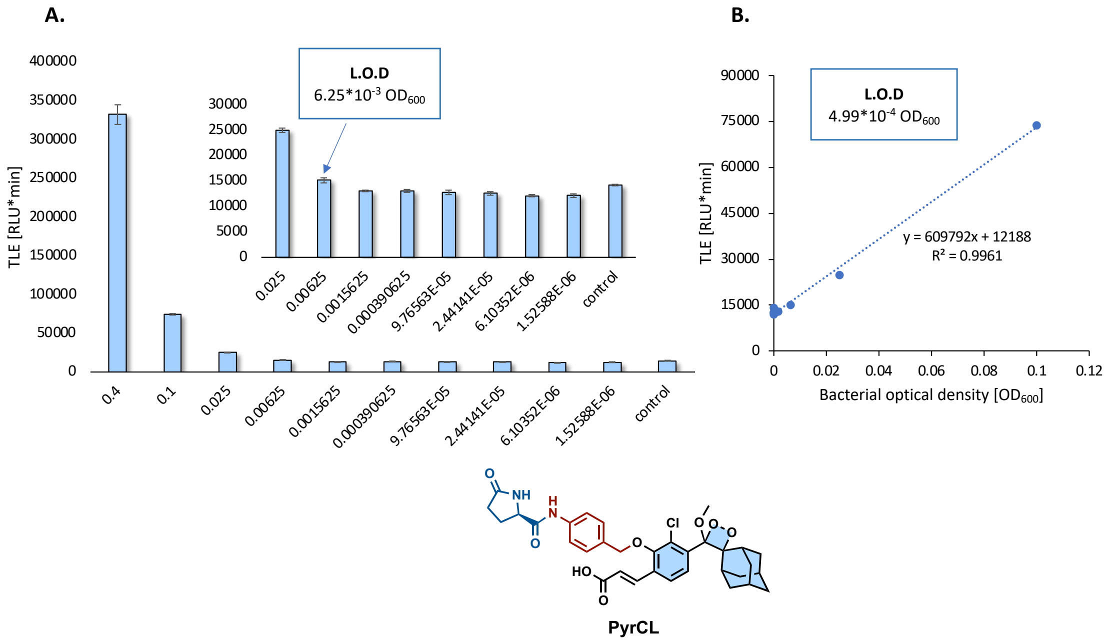

Figure S16. Total light emitted **(A.)** and linear calibration curve **(B.)** after 90 min of PyrCL probe [10 µM] with various bacterial optical densities of *P. aeruginosa* ATCC 47085 [OD_{600} 0.4 – 1.52*10^{-6}] in PBS 7.4, 0.1% DMSO, 37°C. The limit of detection (L.O.D) was determined using two methods: the blank + 3SD (standard deviation) method (left), and secondly, by a linear calibration curve. For the latter, the limit of detection is defined as 3 times the standard deviation of the blank divided by the slope of the linear calibration curve (L.O.D = 3σ/k) (right).

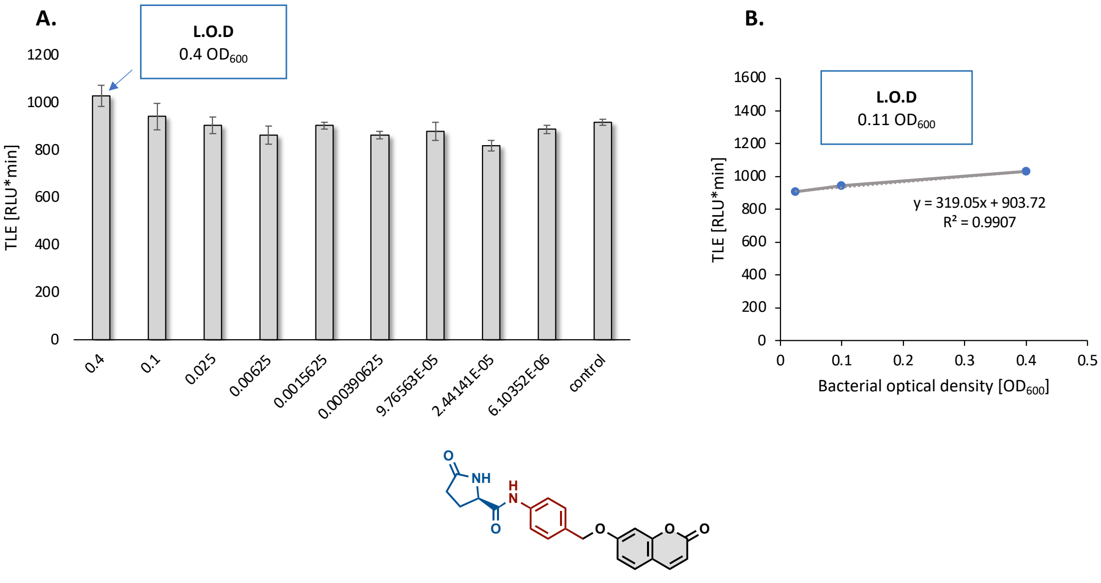

Pyr-HC

Figure S17. Total light emitted **(A.)** and linear calibration curve **(B.)** after 3 hours of Pyr-HC probe [50 µM] with various bacterial optical densities of *P. aeruginosa* ATCC 47085 [OD_{600} 0.4 – 6.10*10^{-6}] in PBS 7.4, 0.1% DMSO, 37°C. $\lambda_{ex} = 350nm$.

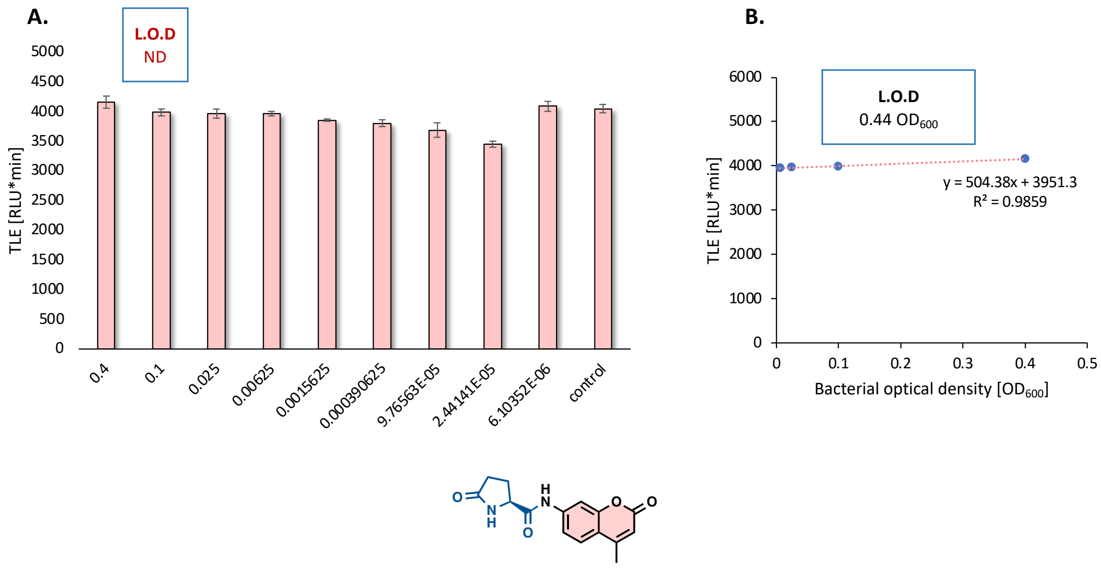

Pyr-AMC

Figure S18. Total light emitted **(A.)** and linear calibration curve **(B.)** after 3 hours of Pyr-AMC probe [50 µM] with various bacterial optical densities of *P. aeruginosa* ATCC 47085 [OD_{600} 0.4 – 6.10*10^{-6}] in PBS 7.4, 0.1% DMSO, 37°C. $\lambda_{ex} = 350nm$.

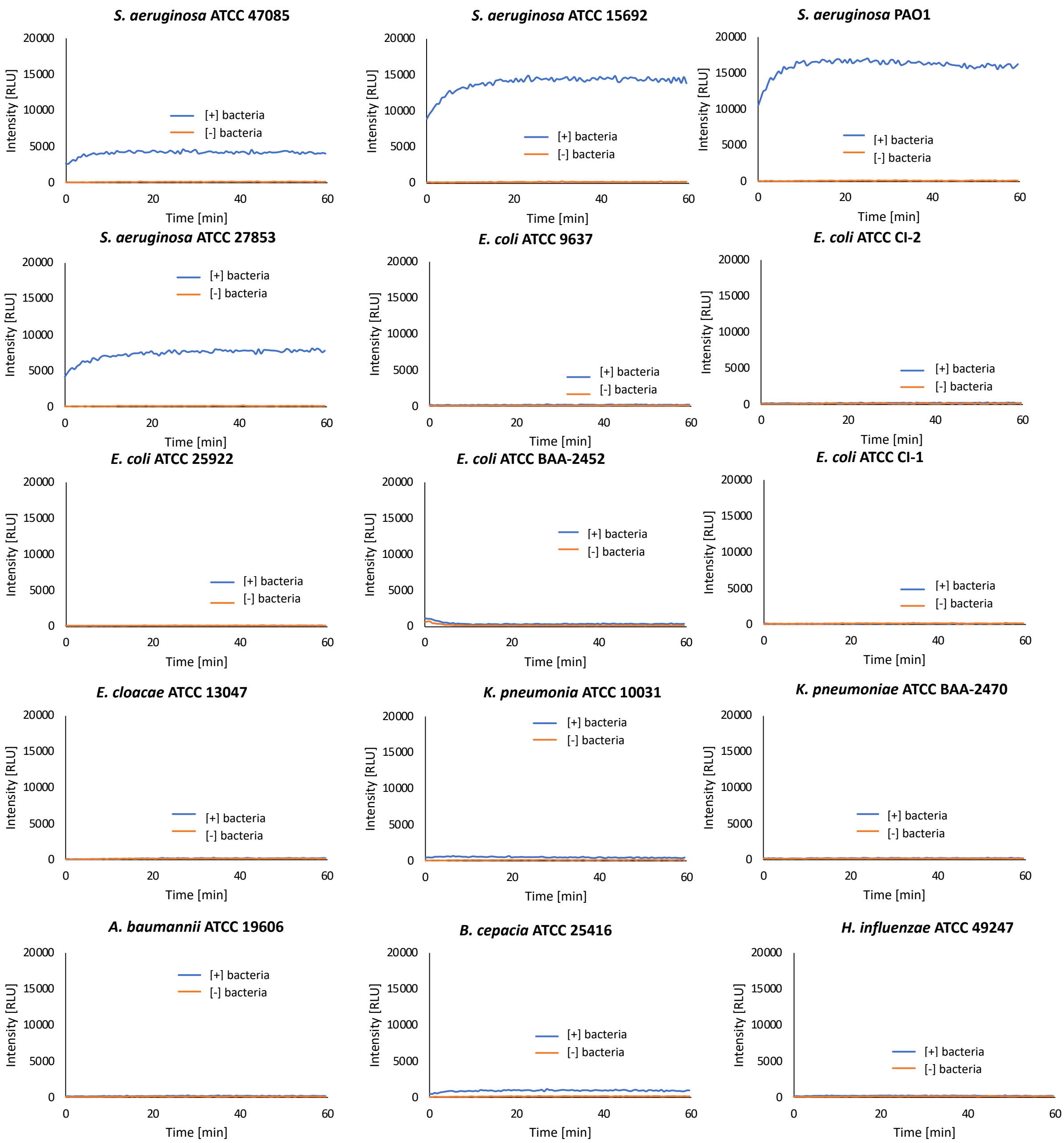

Figure S19. Chemiluminescent kinetic profiles of PyrCL probe [10µM] the 15 strains of gram-negative bacteria, [OD_{600} 0.4], in PBS (pH 7.4), 0.1% DMSO, 37°C.

NMR spectra

Compound 1

^{1}H-NMR

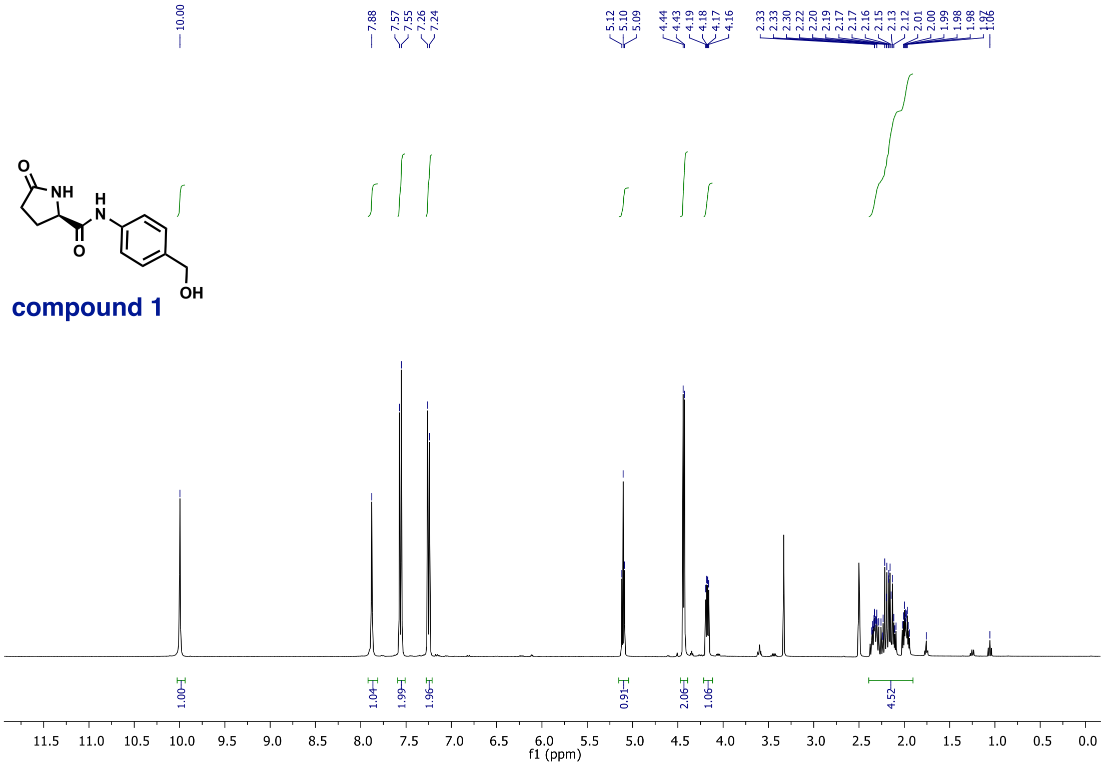

^{13}C-NMR

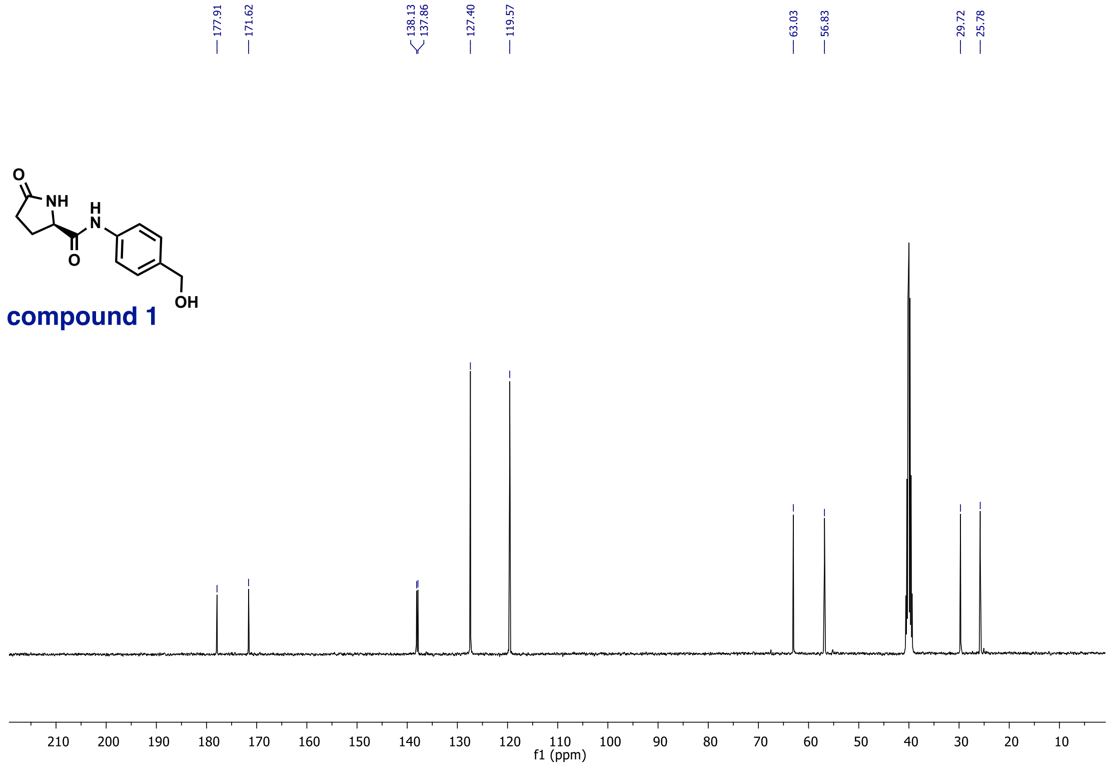

Compound 2

^{1}H-NMR

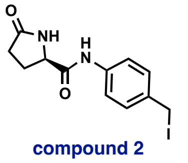

compound 2

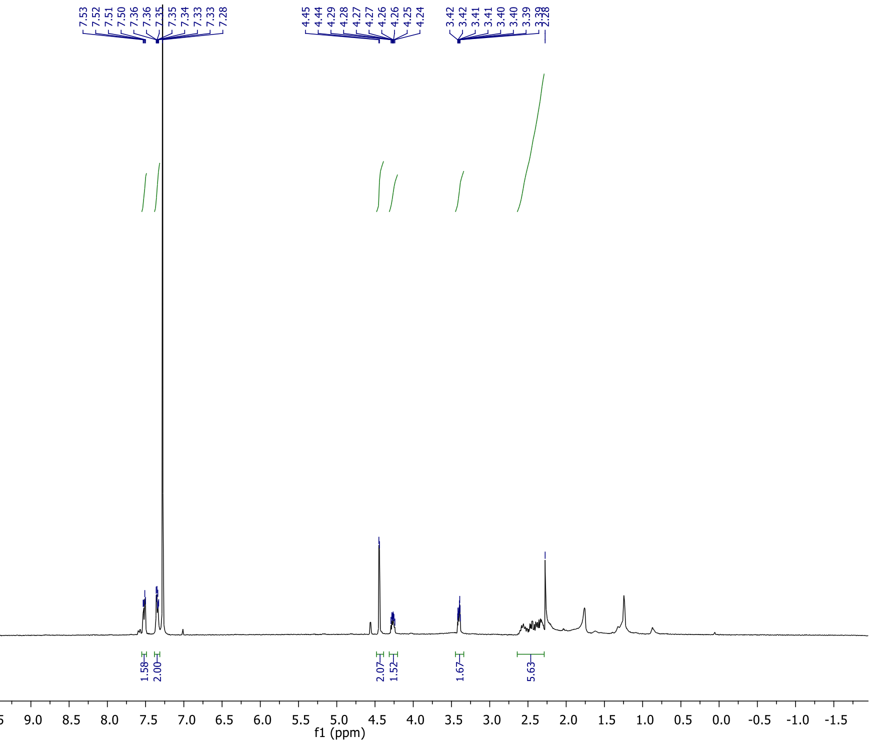

^{13}C-NMR

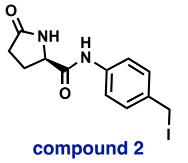

compound 2

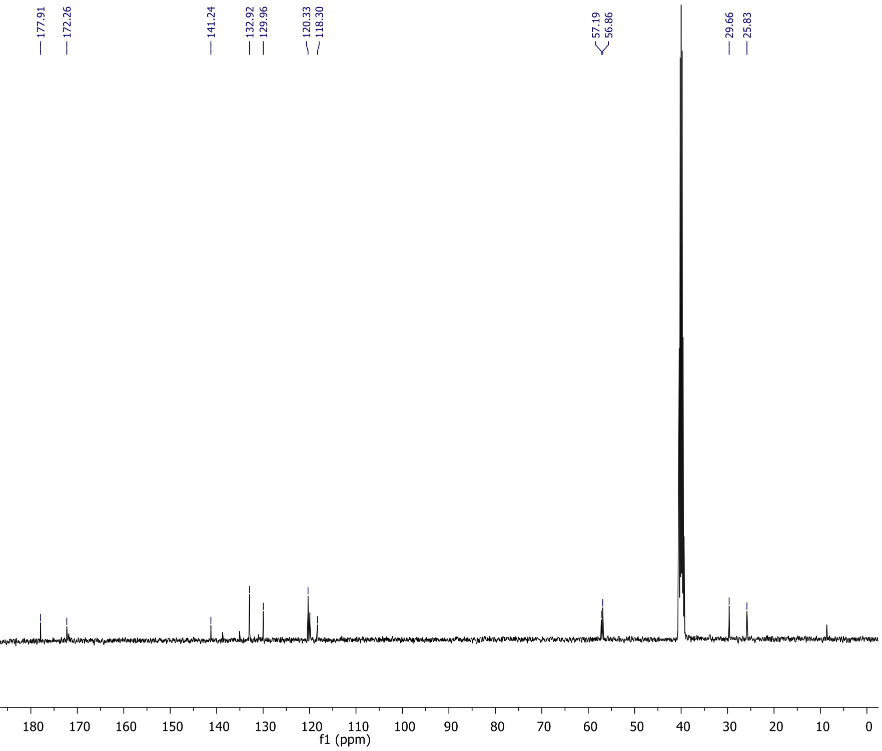

Compound 3

^{1}H-NMR

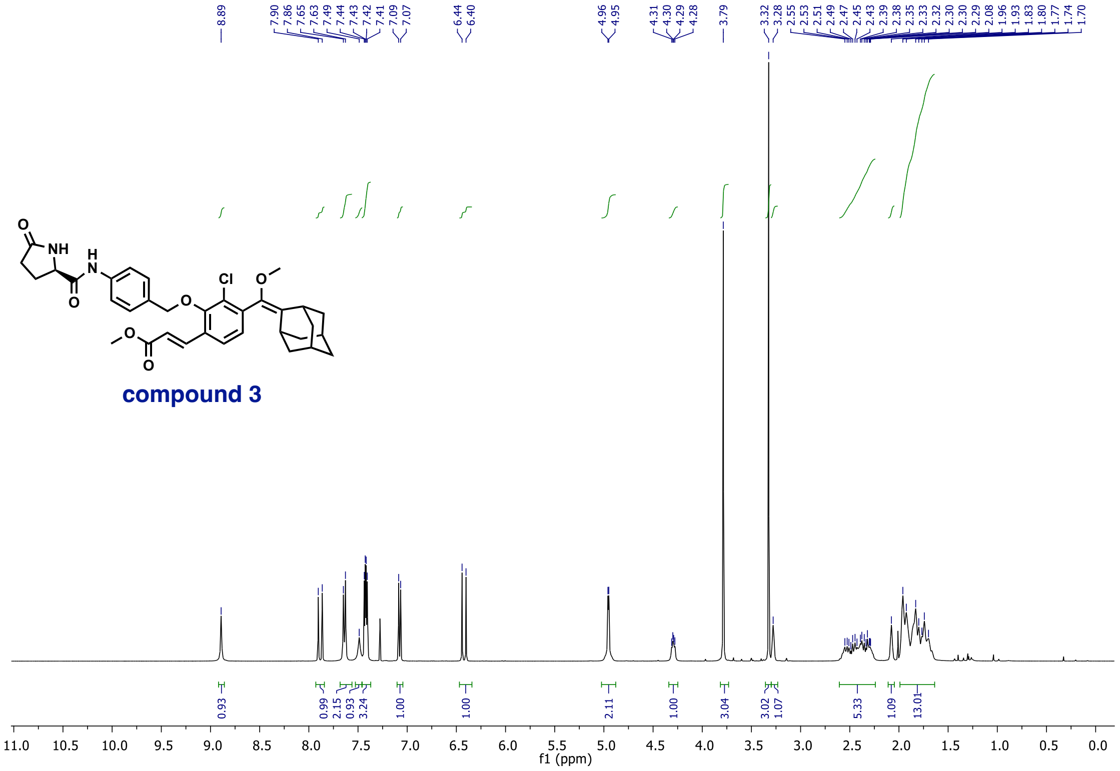

^{13}C-NMR

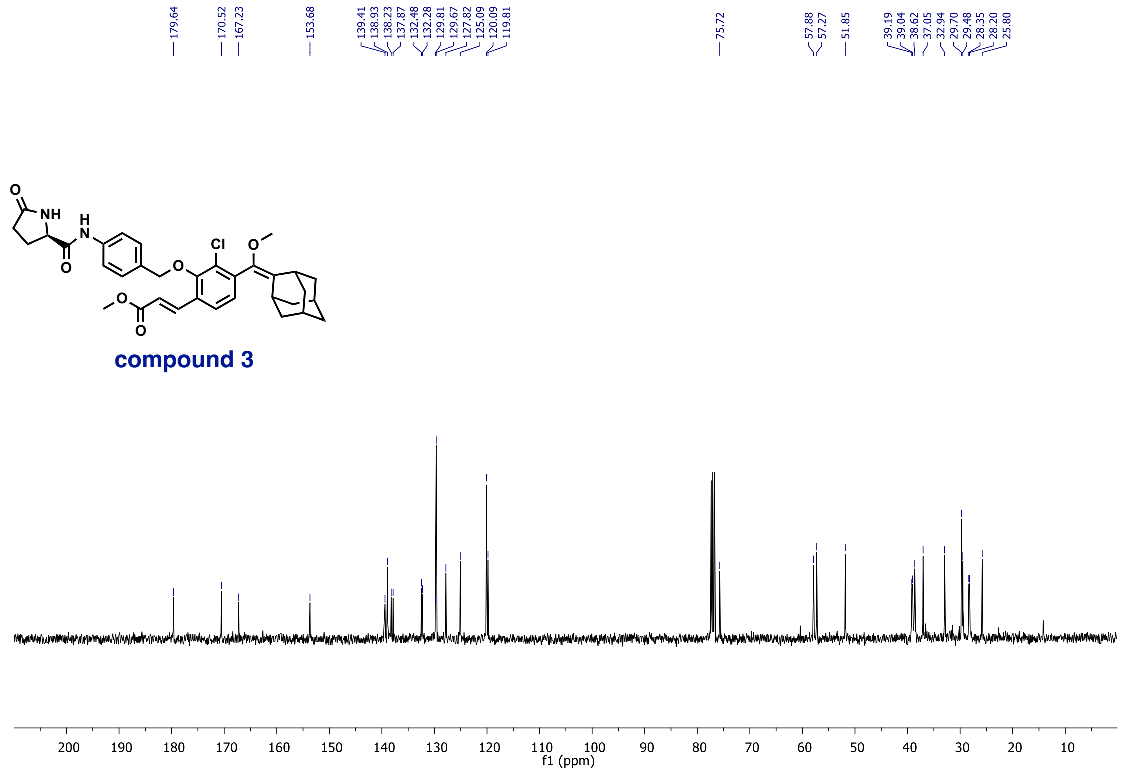

Compound 4

^{1}H-NMR

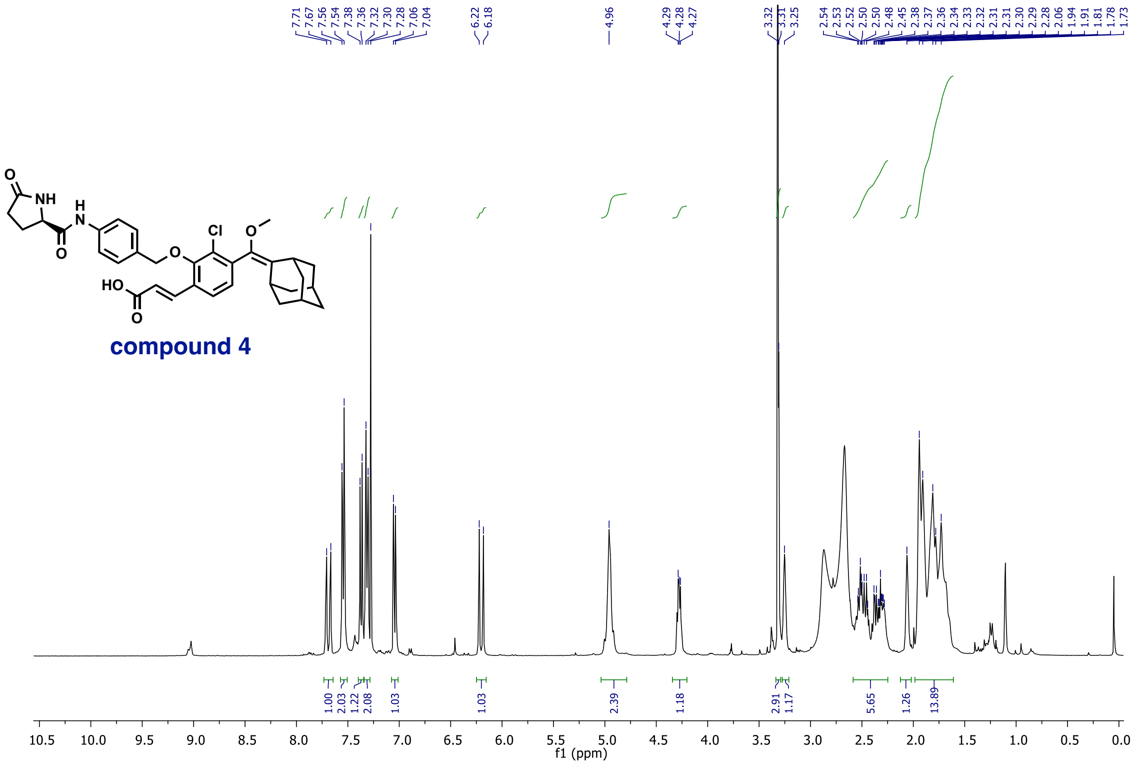

compound 4

^{13}C-NMR

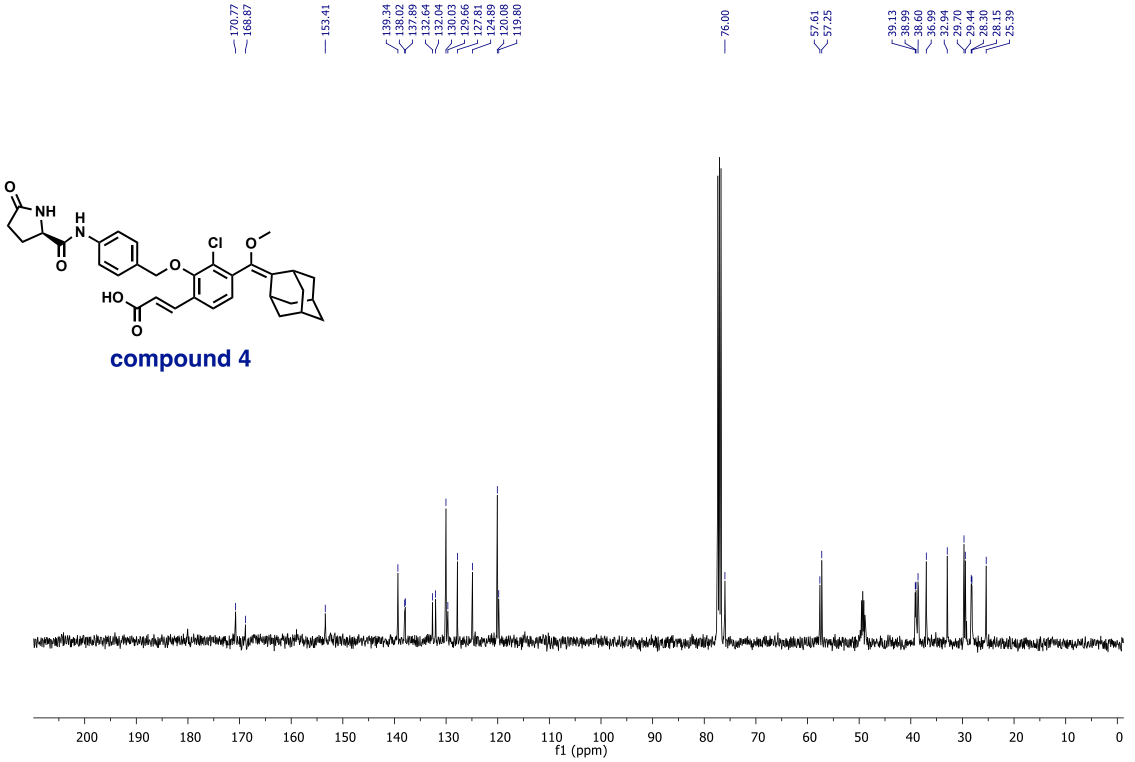

compound 4

Probe PyrCL

^{1}H-NMR

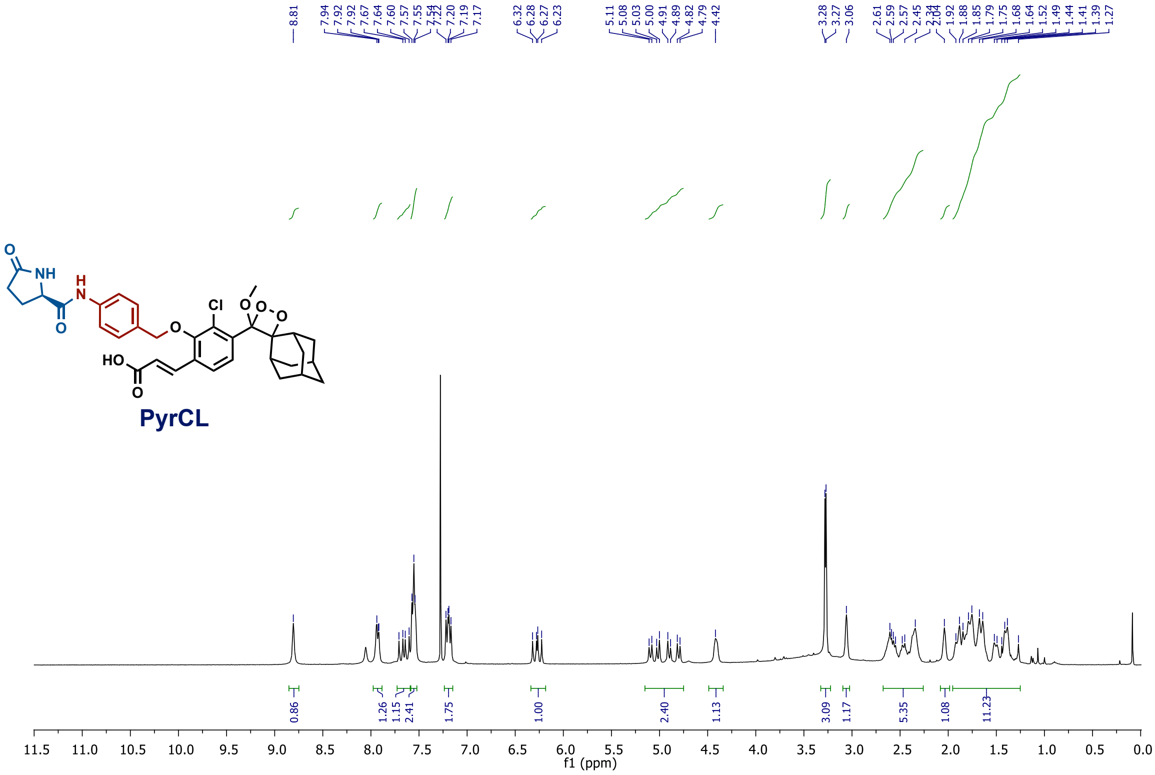

^{13}C-NMR

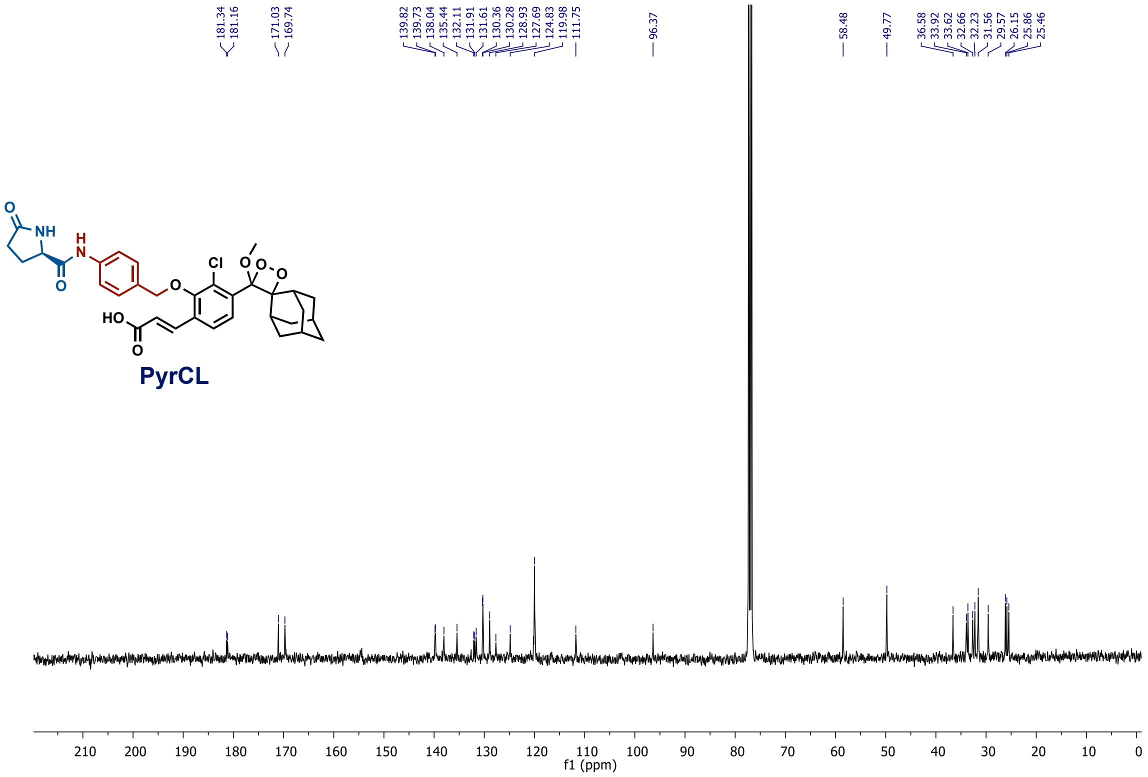

Probe Pyr-HC

^{1}H-NMR

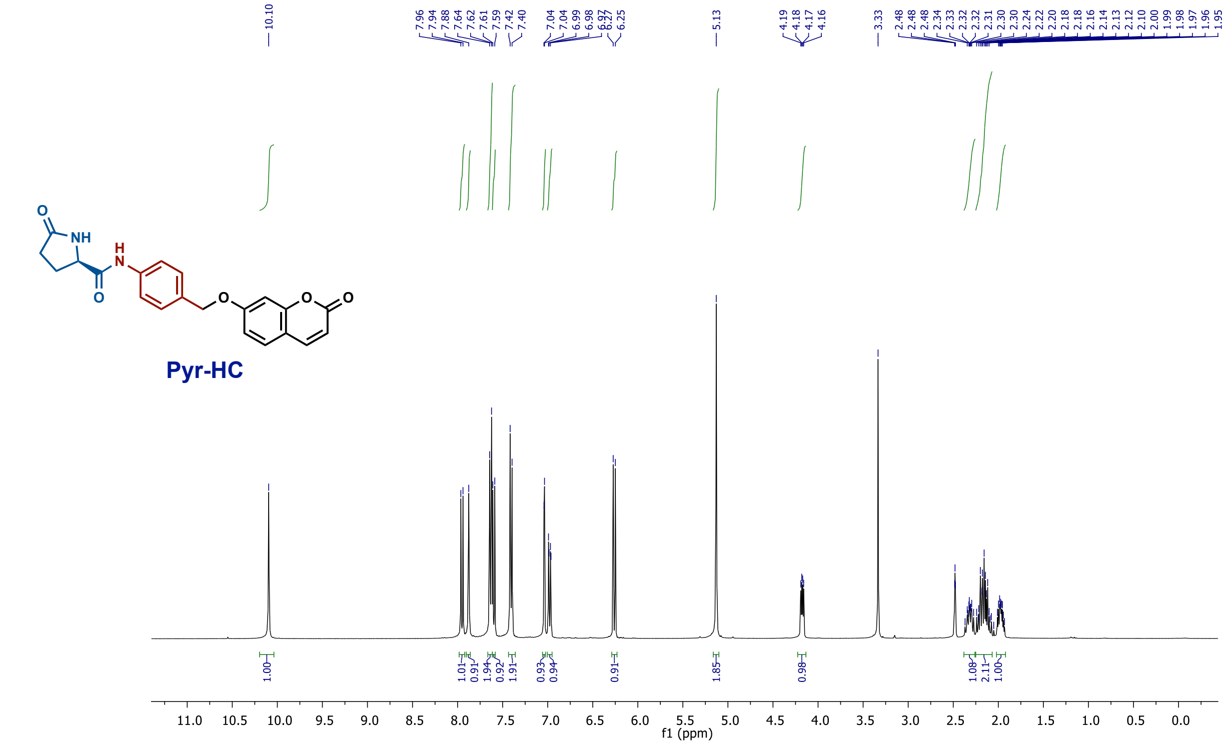

^{13}C-NMR

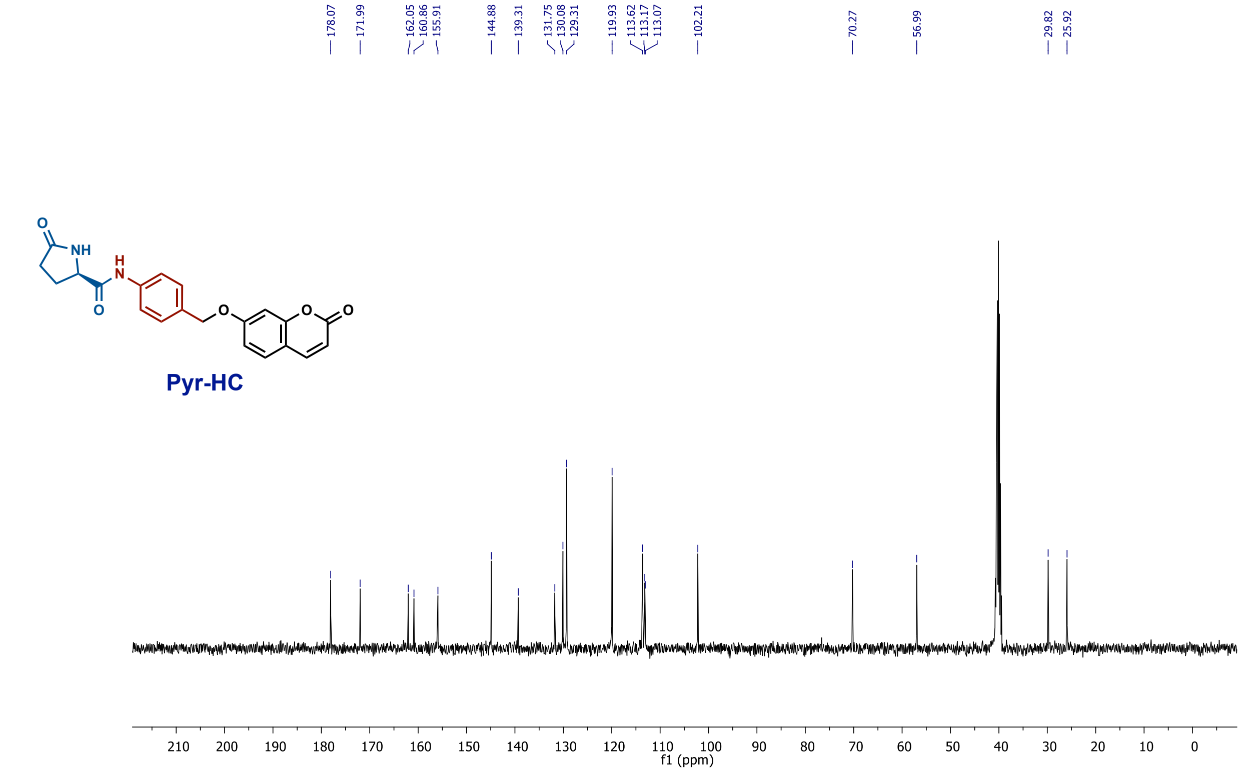

HPLC spectra of key compounds

Compound 3

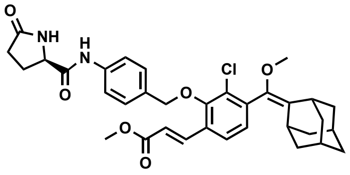

compound 3

3D HPLC spectra (50-100% ACN in water, 0.1%TFA)

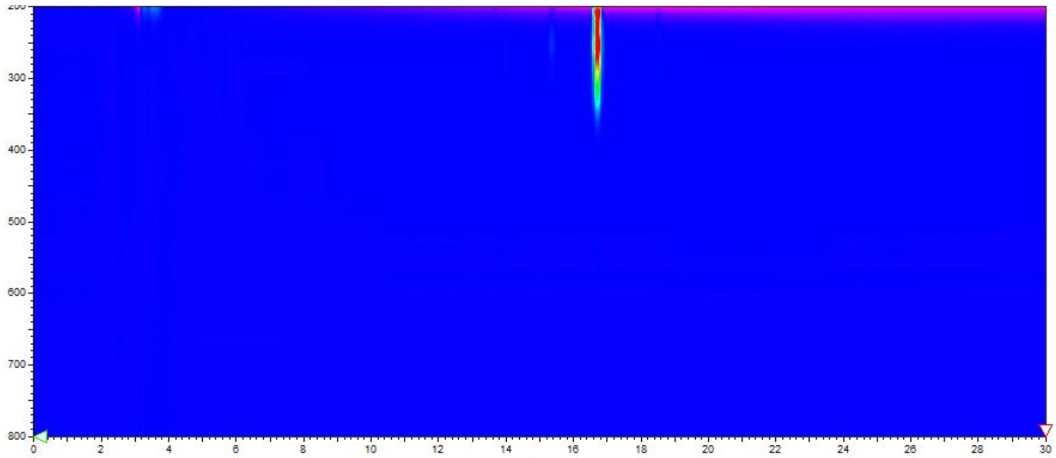

2D HPLC spectra (Absorbance measured at 280nm)

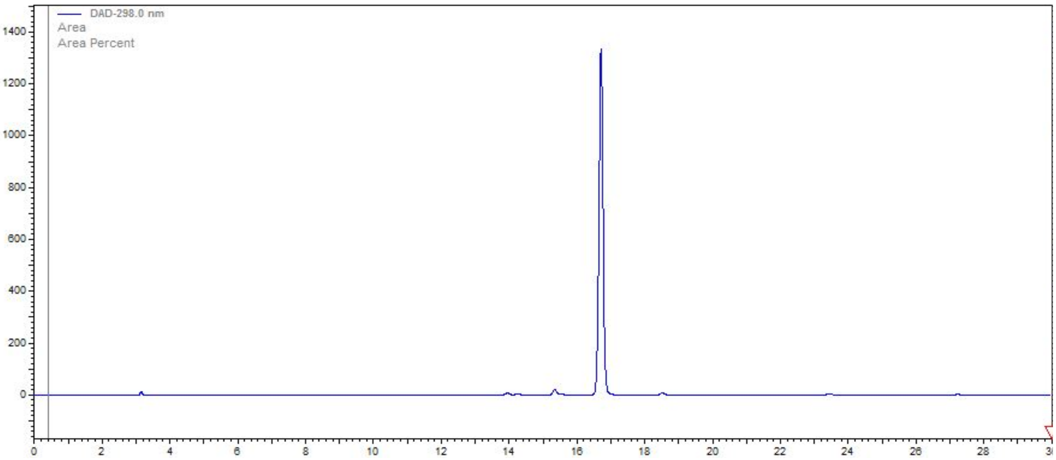

Compound 4

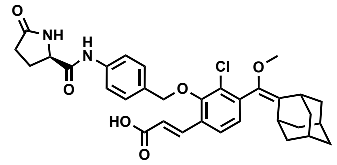

compound 4

3D HPLC spectra (70-100% ACN in water, 0.1%TFA)

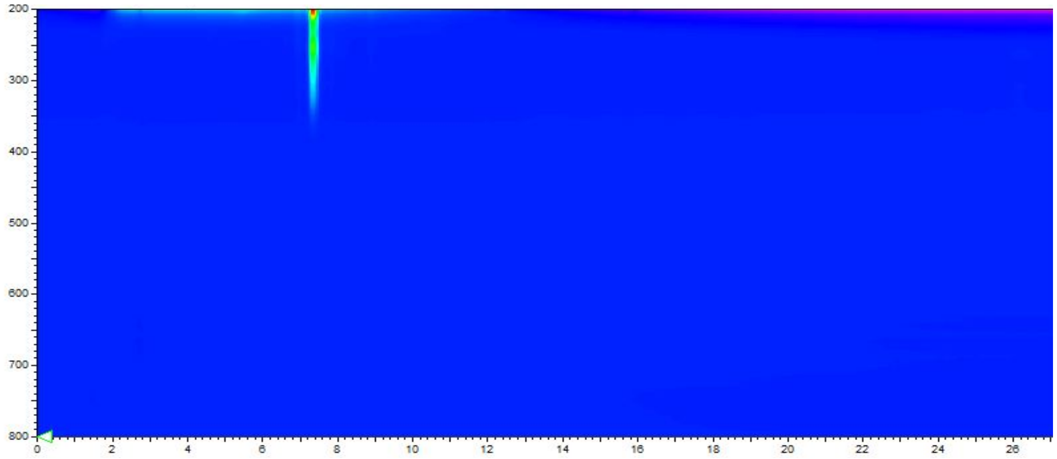

2D HPLC spectra (Absorbance measured at 280nm)

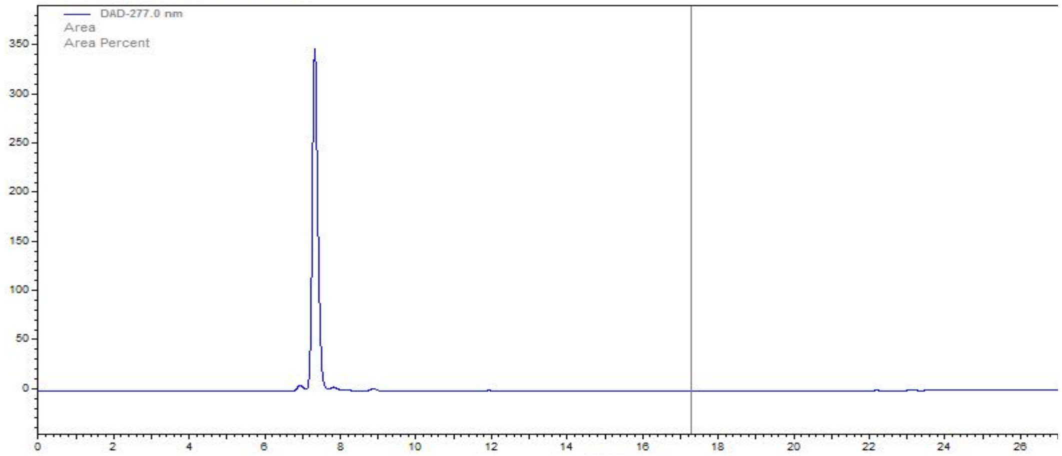

Probe PyrCL

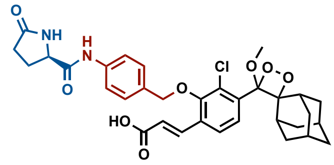

3D HPLC spectra (70-100% ACN in water, 0.1%TFA)

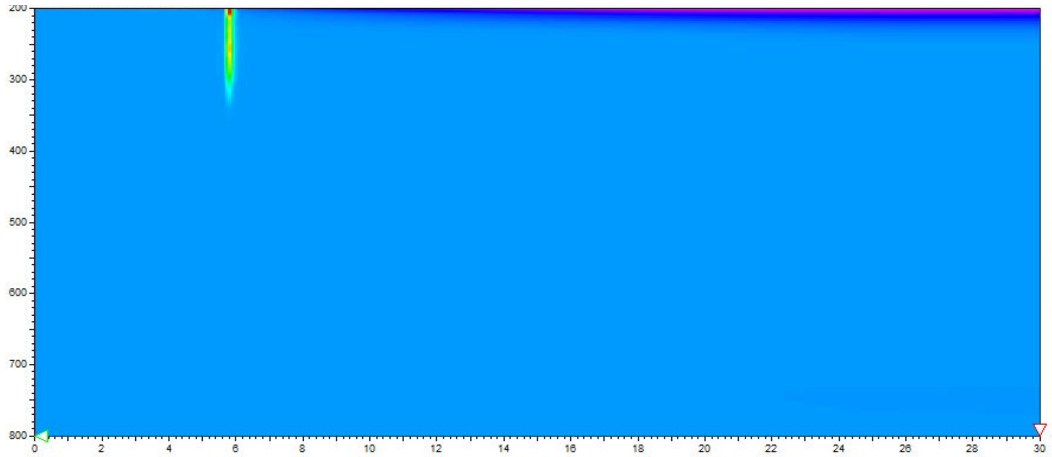

2D HPLC spectra (Absorbance measured at 280nm)

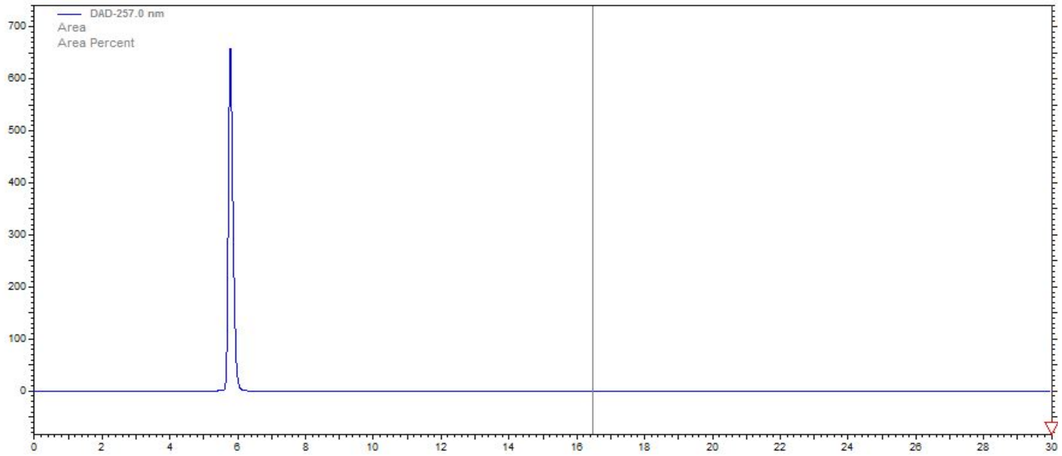

Probe Pyr-HC

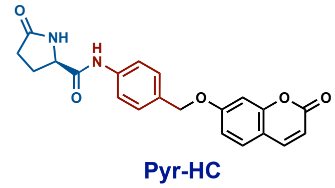

3D HPLC spectra (30-100% ACN in water, 0.1%TFA)

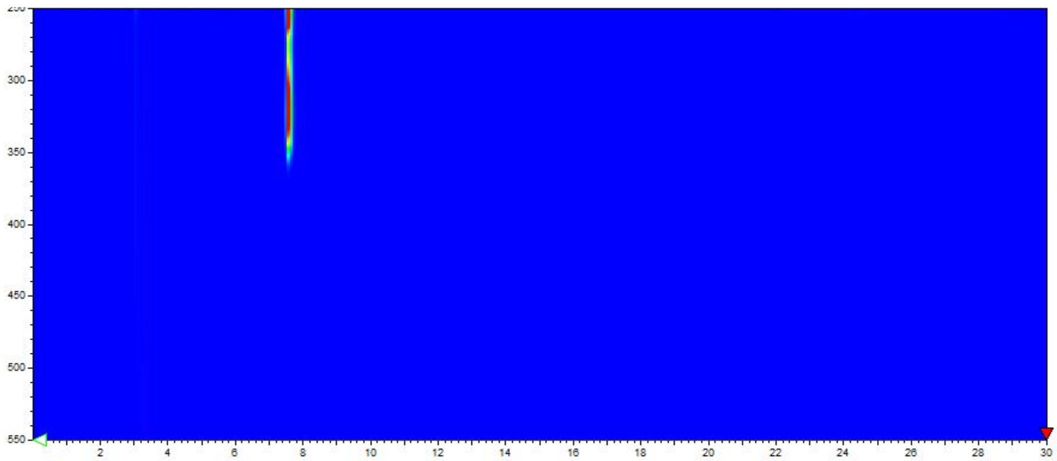

2D HPLC spectra (Absorbance measured at 320nm)

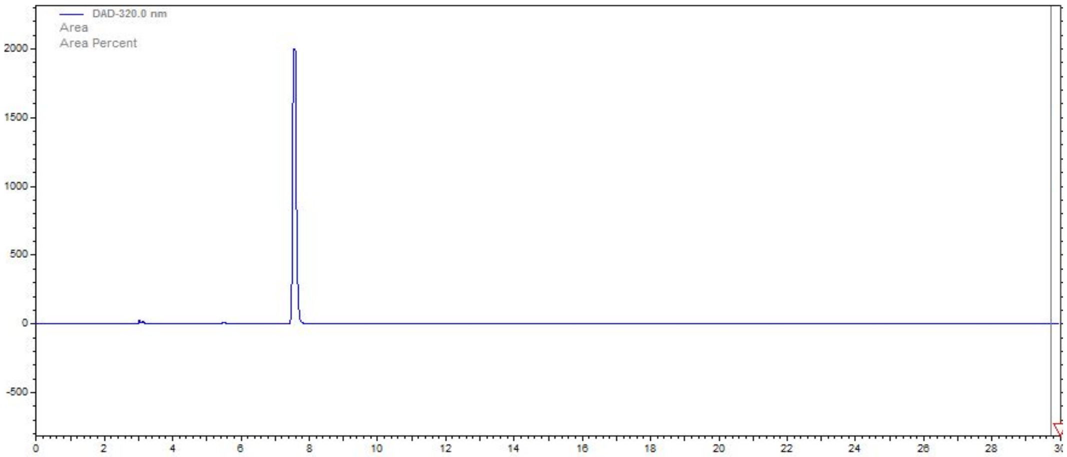

References

(1) Shelef, O.; Kopp, T.; Tannous, R.; Jospe-Kaufman, M.; Arutkin, M.; Reuveni, S.; Shabat, D.; Fridman, M. Enzymatic Activity Profiling Using an Ultra-Sensitive Array of Chemiluminescent Probes for Bacterial Classification and Characterization. *J. Am. Chem. Soc.* **2024**, Article ASAP. (accessed 2024-02-2).

(2) Hananya, N.; Reid, J. P.; Green, O.; Sigman, M. S.; Shabat, D. Rapid chemiexcitation of phenoxy-dioxetane luminophores yields ultrasensitive chemiluminescence assays. *Chem. Sci.* **2019**, *10* (5), 1380-1385.

(3) El-Telbany, M.; El-Didamony, G.; Askora, A.; Ariny, E.; Abdallah, D.; Connerton, I. F.; El-Shibiny, A. Bacteriophages to Control Multi-Drug Resistant Infection of Dental Root Canals. *Microorganisms* **2021**, *9* (3), 517.

(4) Giard, J. C.; Rince, A.; Capiaux, H.; Auffray, Y.; Hartke, A. Inactivation of the stress- and starvation-inducible *gls24* operon has a pleiotrophic effect on cell morphology, stress sensitivity, and gene expression in *Enterococcus faecalis*. *J. Bacteriol.* **2000**, *182* (16), 4512-4520.
